# Application of BMP in Bone Tissue Engineering

**DOI:** 10.3389/fbioe.2022.810880

**Published:** 2022-03-31

**Authors:** Liwei Zhu, Yuzhe Liu, Ao Wang, Zhengqing Zhu, Youbin Li, Chenyi Zhu, Zhenjia Che, Tengyue Liu, He Liu, Lanfeng Huang

**Affiliations:** ^1^ Department of Orthopedics, The Second Hospital of Jilin University, Changchun, China; ^2^ Orthopaedic Research Institute of Jilin Province, Changchun, China

**Keywords:** bone morphogenetic protein, BMP, biomaterials, bone tissue engineering, bone healing

## Abstract

At present, bone nonunion and delayed union are still difficult problems in orthopaedics. Since the discovery of bone morphogenetic protein (BMP), it has been widely used in various studies due to its powerful role in promoting osteogenesis and chondrogenesis. Current results show that BMPs can promote healing of bone defects and reduce the occurrence of complications. However, the mechanism of BMP *in vivo* still needs to be explored, and application of BMP alone to a bone defect site cannot achieve good therapeutic effects. It is particularly important to modify implants to carry BMP to achieve slow and sustained release effects by taking advantage of the nature of the implant. This review aims to explain the mechanism of BMP action *in vivo*, its biological function, and how BMP can be applied to orthopaedic implants to effectively stimulate bone healing in the long term. Notably, implantation of a system that allows sustained release of BMP can provide an effective method to treat bone nonunion and delayed bone healing in the clinic.

## 1 Introduction

Bone morphogenetic proteins (BMPs) were originally discovered to promote bone growth in muscle. Since 1965, when Dr. Urist discovered that BMP can induce bone growth in muscle (Urist, 1965), multiple subsequent studies have demonstrated that BMPs function in a large variety of physiological and pathological processes. BMP is an important member of the transforming growth factor-beta (TGF-β) superfamily, a group of highly conserved homologous signalling proteins that play an important role in embryogenesis, organogenesis, cell proliferation, and stem cell differentiation(Miyazawa et al., 2002). Therefore, BMPs have been applied in a plethora of tissues and organs. To date, approximately 20 BMP family members have been identified and characterized. BMP is a dimeric molecule composed of two polypeptide chains linked by a single disulfide bond. According to the structural similarity of BMP amino acid sequences, BMP family members are generally divided into four categories: BMP2/4; BMP5/6/7/8; BMP9/10; and BMP12/13/14 (Liu et al., 1995; Gomez-Puerto et al., 2019).

In orthopaedics, BMPs are naturally secreted multifunctional proteins that play crucial roles throughout the developing skeletal system. BMPs have been proven to be key factors with significant osteogenic functions, regulating bone balance by controlling the differentiation of osteoblasts and osteoclasts (Poynton and Lane, 2002; Lavery et al., 2008). Notably, BMP-2 and BMP-7 can significantly enhance osseointegration (Dent-Acosta et al., 2012; Dolanmaz et al., 2015). Therefore, the Food and Drug Administration (FDA) has approved two factors containing recombinant human BMP (rhBMP)-2 and rhBMP-7 for a few orthopaedic disease treatments, such as open fractures, non-union fractures, vertebral fusion, and maxillofacial bone enhancement (Cecchi et al., 2016).

Fracture is a serious public health problem and is one of the diseases that most affects people’s quality of life. It brings a heavy burden to patients and the medical system. Fracture also causes pain and affect a patient’s exercise ability. If recovery is improper, it is very likely to reduce life-long mobility. Fracture healing is a very robust repair process that depends on many factors, including fracture type, anatomical site, and associated trauma to soft tissue and vascular structures (Hackl et al., 2017). After the fracture heals, the epiphysis can achieve structural stability, even exceeding the stability of the unfractured bone, and then undergo callus remodelling, restoring the geometry and function of the original bone. Generally, the fracture can be cured after reduction and fixation, but in some cases, severe bone defects or nonunion occur. Generally, most fracture patients will heal within a few weeks to a few months. Although most fracture patients heal quickly after timely treatment, a small number of patients will have delayed fracture union or fracture nonunion, especially patients with larger open bone injuries and non-union (Hissnauer et al., 2017). At this time, the traditional treatment method has little effect. Due to the special nature of fracture healing and fixation, the current standard of treatment for fractures includes reduction and fixation, and the requirements for clinical treatment are as follows: 1) speed up fracture healing, 2) speed up normal function recovery, and 3) reduce fracture healing complications (Goldhahn et al., 2008).

With the rapid development of orthopaedic implants, such as intramedullary nails, steel plates, joint prostheses, pedicle screws and other materials, the incidence of delayed union or nonunion of fractures has been greatly reduced when treating fractures or nonunions, shortening the healing time and reducing fixation-related complications (von Ruden et al., 2016). Notably, some patients still suffer from complications, and traditional treatment methods often cannot guarantee that the fracture will reach the complete clinical cure standard. Although device-assisted therapy is still an important part of fracture treatment, for example, reduction and fixation of the fracture site through intramedullary needles, screws, or metal plates, implants may prolong the fracture healing time and cause infection and chronic pain. Importantly, exploring new treatments for fractures has become the focus of orthopaedic research (Annis et al., 2015).

The FDA has approved rhBMP-2 and rhBMP-7 for orthopaedic treatments, such as open fractures, nonunion fractures, vertebral fusion, and maxillofacial bone enhancement (Cecchi et al., 2016). In recent years, the application of growth factors may bring new breakthroughs in the treatment of fractures. Depending on the morphology of the bone defect, rhBMP-2 and rhBMP-7 can be used in combination with bone substitutes or collagen sponges to achieve better results (Dent-Acosta et al., 2012). Importantly, with the increase in the number of treatments, retrospective studies have provided more data to analyse whether BMP can promote fracture healing. Current research advances indicate that application of biological agents may lead to significant progress in improving treatment effects.

At present, many biomaterials carrying BMP have been used in orthopaedic treatments and have achieved desired results. These implant materials should have good mechanical properties, excellent biocompatibility, good corrosion resistance, high wear resistance, and osteointegration capability (Han et al., 2014; Chen et al., 2020). Whether implant materials are compatible with surrounding tissues and whether they can promote bone formation is a focus of orthopaedic research (Sul et al., 2002; Geetha et al., 2009). The application of BMPs on scaffolds for clinical treatment of orthopaedic diseases has initially achieved a certain effect. To achieve better therapeutic effects, people have improved biomaterials with high biocompatibility to make their design and manufacturing more perfect, which can continuously and effectively promote bone formation. This research method of bone tissue engineering shall receive more and more attention (Bosemark et al., 2015; Ding et al., 2016; Huang et al., 2018). To date, extensive research has focused on combining new biomaterials and exploring new material synthesis technologies, which can not only improve the success of surgery but also reduce the cost of surgery (Wang L. et al., 2019; Zhang X. et al., 2019). From what has been discussed above, biomaterial-loaded BMPs have become research hotspots and open up new prospects for orthopaedic disease research and treatment.

In this review, we summarize recent research advances in BMP functions in orthopaedics, including biological functions, applications in bone tissue engineering and applications in common clinical diseases.

## 2 Advanced Research Progress on the Biological Function of BMPs in the Field of Orthopaedics

**FIGURE 1:**
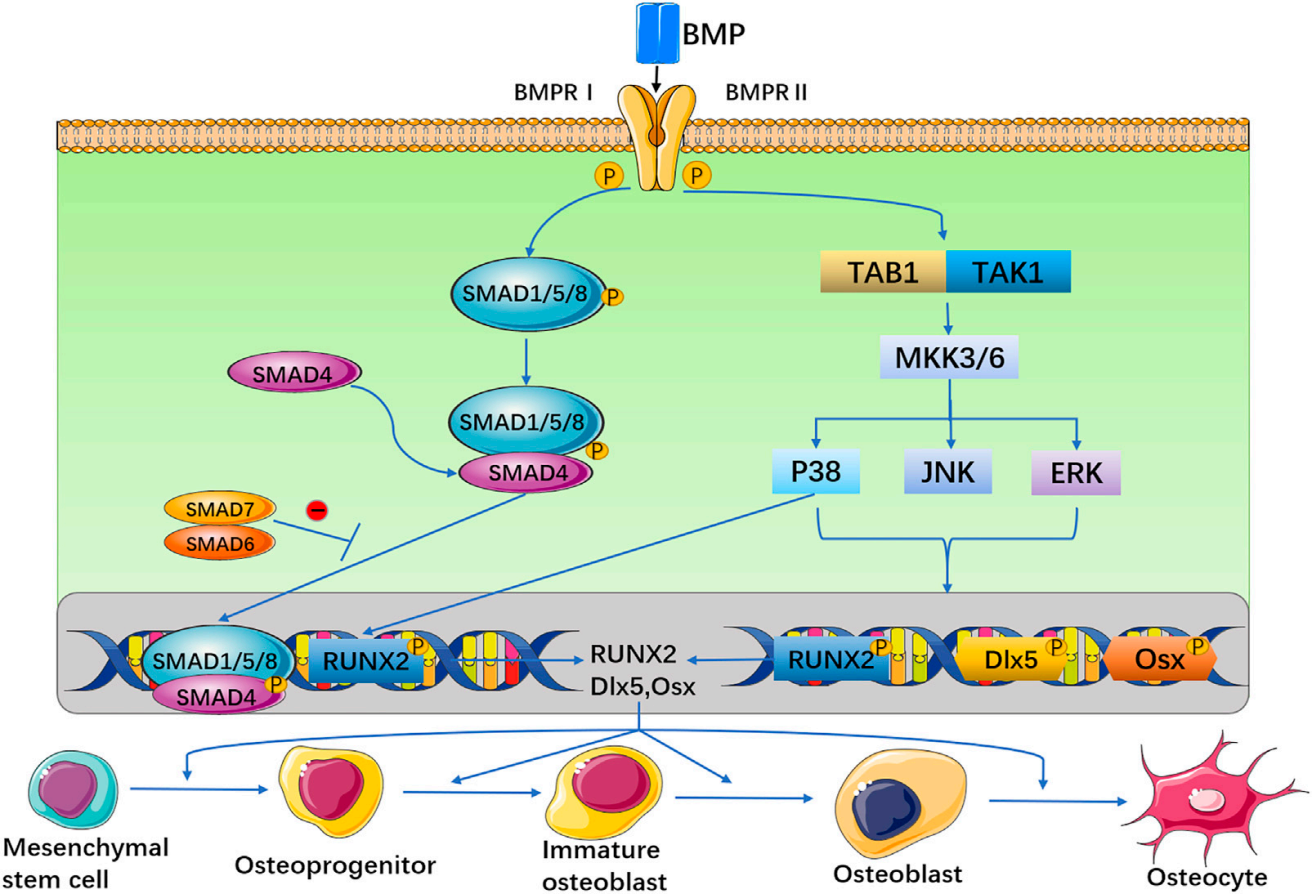
The BMP signal is transduced through BMPRI and BMPRII receptors. These two receptors are combined into a functional complex, to initiate further signaling pathways. On the one hand, activated BMP type I receptor phosphorylates Smad-dependent signaling pathways. On the other hand, BMP receptors activate non-Smad-dependent signaling pathways, that is, activate p38 MAPK, JNK, and ERK signaling pathways. Then, BMP can stimulate the expression of three osteogenic main transcription factors Runx2, D1x5 and Osx.

Together, the biological functions of BMP regulate the growth and development of organisms through different signalling pathways in cells. BMP signalling is transduced through serine (Ser)/threonine (Thr) protein kinase receptors, namely, type I receptors (BMPRIs) and type II receptors (BMPRIIs) (Liu et al., 1995; Gomez-Puerto et al., 2019). These two receptor types are combined into a functional complex to initiate further signalling pathways (Koenig et al., 1994). The BMP ligand activates BMPRII, and then, BMPRII phosphorylates BMPRI. Activated BMPRI recruits and phosphorylates Smad-dependent and non-Smad-dependent signalling pathways and then transduces signals into the nucleus and controls osteogenic gene expression. On the one hand, activated BMPRI phosphorylates Smad-dependent signalling pathways, including Smad 1, Smad 5, and Smad 8 (Yamamoto et al., 1997; Nishimura et al., 1998; Kaneko et al., 2000; Pera et al., 2004), and then combines with Smad 4 to form a hybrid complex, which is transported to the nucleus and regulates target gene transcription. Smad 6 or 7 negatively regulates Smad signalling by preventing receptor-activated Smad (R-Smad) phosphorylation, inhibits the expression of osteoblast-related genes and blocks the differentiation of osteoblasts. On the other hand, BMP receptors activate non-Smad-dependent signalling pathways, namely, the p38 mitogen-activated protein kinase (MAPK), extracellular signal-regulated kinase (ERK) and c-Jun N-terminal kinase (JNK) signalling pathways (Guicheux et al., 2003). Then, BMP signalling can stimulate the expression of the main osteogenic transcription factors runt-related transcription factor 2 (Runx2), distal-less homeobox 5 (Dlx5), and osterix (Osx) (Lee et al., 2003), and Runx2 plays a key role in the induction of osteogenesis (Figure 1) (Celil and Campbell, 2005). In addition, TGF-β, Wnt, Hedgehog, Notch, fibroblast growth factor (FGF), and other signalling pathways also interact with the BMP signalling pathway (Wang N. et al., 2017; Zhang L. et al., 2019). At the cellular level, BMP exists as a ligand of receptors on the membrane of various cells, such as osteoblasts, osteoclasts, adipose stem cells, mesenchymal stem cells, and tendon fibroblasts, through concentration gradient diffusion. When the receptors of these cells are activated, they induce cells to differentiate and proliferate (Rawadi et al., 2003; Wu et al., 2016). Therefore, BMPs play an important role in the growth and development of bones and in homeostasis of the bone environment effect.

### 2.1 Cartilage Formation

Articular cartilage is often damaged by mechanical wear, inflammation, and external forces. Due to the nonvascular nature of cartilage tissue, once articular cartilage is damaged, its ability to spontaneously heal and regenerate is very weak, which reduces joint motor function. In addition, cartilage formation may also be critical for initial bridging and subsequent stabilization of a fracture and can reduce the occurrence of late complications. Compared to osteogenesis alone, timely stimulation of cartilage in fractured epiphyses may be a more effective way to prevent delayed bone healing or nonunion of the bone (Shum et al., 2003; Hatakeyama et al., 2004; El-Magd et al., 2013; Kostenuik and Mirza, 2017; Wang T. et al., 2019; Yang et al., 2021). Therefore, cartilage formation plays a crucial role in the skeletal system. Currently, the use of tissue grafts to repair articular cartilage and other connective tissue in the joint has limited therapeutic effects. How to effectively repair and regenerate joints presents great challenges in current research.

Cartilage formation is a multistep process. At the gene level, the expression of osteogenic and cartilage-related genes is dependent on the BMP signalling pathway. Moreover, the BMP signalling pathway can directly regulate the expression of Runx2 (Akiyama et al., 2002). Runx2 is the key gene in the bone marrow mesenchymal stem cell (BMSC) differentiation process, regulating the expression of Osx, sry-related HMG-box (Sox) 9, type II collagen, aggrecan (Acan), and other genes. BMSCs form chondrocytes by maintaining Runx2 expression (Pini et al., 2018). By measuring the expression levels of BMPs in normal human cartilage, BMP-2, BMP-4, BMP-5, BMP-6, BMP-7, and BMP-9 have been found to be highly expressed in cartilage tissue (Suttapreyasri et al., 2006), suggesting that a proper proportion of BMPs may maintain normal homeostasis in normal human cartilage tissue. These BMPs have a precise impact on cartilage formation *in vivo*, but the relationship between the expression of a single BMP type and cartilage formation requires more careful *in vivo* and *in vitro* studies.

BMP-2 increases the expression level of Runx2 at the transcriptional and posttranscriptional level by inhibiting the expression of cyclin-dependent kinase 4, ubiquitination of Runx2 and protease degradation (Shu et al., 2011). BMP-2 induces Sox9 expression, which in turn stimulates the expression of cartilage markers, such as collagen type II alpha 1 chain (Col2a1) (Zhao C. et al., 2017). BMP-4 plays a guiding role in cartilage formation. BMP-4 can not only induce mesenchymal cells to differentiate into cartilage but can also induce the expression of nodular interstitial cells, thus promoting the formation and maturation of cartilage nodules (Hatakeyama et al., 2004). BMP-5 can increase the expression of hypertrophy markers, type Ⅰ collagen, type Ⅱ collagen, and type X collagen (Guenther et al., 2008; Snelling et al., 2010). In one study, knockout of the BMP-6 gene in chondrocytes leads to a significant decrease in the expression of type II collagen and type X collagen (Ye et al., 2019). BMP-7 can promote the expression of cartilage markers, such as collagen type I alpha 2 chain (Col1a2), Col2a1, collagen type X alpha 1 chain (Col10a1), matrix metalloprotein (mmp) 13, Runx2 and Acan (Yan et al., 2018; Muller et al., 2019). In addition, BMP-9 can increase the expression of type II collagen, Sox 9, Acan and cartilage oligomeric protein in BMSCs (Wang et al., 2014; van Caam et al., 2015; Ren et al., 2016). Because of the overlapping biological functions of different BMPs, some combinations of BMPs may be more effective in cartilage or bone regeneration than a single BMP. Table 1 lists the functions of BMPs in cartilage formation.

**TABLE 1:** Functions of BMPs in cartilage formation.

BMP(s)	Related GENE(s)	Biological function(s)	References
BMP-2	Runx2, Sox5, Sox9, Acan, and Col2a1	Increases the expression level of Runx2, Promote chondrogenesis	Wang T. et al. (2019)
BMP-4	Sox9, type Ⅱ collagen, and type X collagen	Plays a guiding role in cartilage formation. Regulation development of vertebral cartilage, pedicle of vertebral arch and proximal rib	Shum et al. (2003), Hatakeyama et al. (2004), El-Magd et al. (2013)
BMP-5	Type Ⅰ collagen, type Ⅱ collagen, and type X collagen	Promote cartilage formation	Guenther et al. (2008), Snelling et al. (2010)
BMP-6	Type II collagen and type X collagen	Affects cartilage development	Ye et al. (2019)
BMP-7	Col1a2, Col2a1, Col10a1, mmp13, Runx2, and Acan	Promote cartilage formation	Muller et al. (2019)
BMP-9	Type II collagen, Sox9, Acan, and ALK-1	Effectively activate Smad pathway, promote chondrocyte differentiation and osteogenic differentiation	Wang et al. (2014), van Caam et al. (2015), Ren et al. (2016)

### 2.2 Bone Formation

There are two forms of bone formation, endomembranous and endochondral. Intramembranous osteogenesis is the differentiation of mesenchymal cells into embryonic connective tissue membranes, and then differentiation into osteoblasts for proliferation and differentiation. The parietal bone, frontal bone and clavicle of the human body occur in this way. Endochondral ossification is first formed from hyaline cartilage to form a cartilage model, which is then replaced by mineralized bone and continues to expand to both ends. Endochondral ossification occurs mainly in long bones, short bones and some irregular bones such as the base of the skull and the back of the skull. Studies have shown that there is no cartilage tissue at an early time point in bone defect healing. Interestingly, the addition of BMP resulted in the early appearance of chondroid tissue. BMP can promote the proliferation and differentiation of chondrocytes, and can guide endochondral ossification, which plays an important role in the development of endochondral bone (Dang et al., 2017; Daly et al., 2018; Klosterhoff et al., 2022). BMP stimulates the first stage of endochondral ossification, including effective stem cell recruitment and cartilage formation. BMP plays an important role in endochondral bone development and has various functions in bone formation, including bone morphogenesis, growth plate development and osteoblast differentiation (Wu et al., 2016; Long et al., 2021; Strong et al., 2021; Yang et al., 2021). Bone remodelling is a physiological process involving absorption of old bone and formation of new bone. These two physiological processes are influenced by systemic regulation and the local release of growth factors, such as BMPs, in the body. During osteoblastic differentiation, BMSCs are controlled by Runx2 and Osx genes, which are considered to be key genes for osteoblastic differentiation. Among them, Runx2 plays a leading role in the entire differentiation process, while the BMP signalling pathway directly affects the expression of Runx2, therefore, BMPs can more effectively induce osteoblast differentiation and regulate bone formation (Yokouchi et al., 1996; Lieberman et al., 1998; Baltzer et al., 2000; Krishnan et al., 2001; Sojo et al., 2005; Bessa et al., 2008; Seo et al., 2014; Teotia et al., 2017; Pini et al., 2018; Ball et al., 2019; Chen et al., 2019; Long et al., 2021).

rhBMP-2 and rhBMP-7 are orthopaedic surgery-induced osteogenic adjuvants approved by the FDA (Cecchi et al., 2016). BMP-2 is an essential endogenous medium for fracture repair (Peng et al., 2005; Tsuji et al., 2006; Song et al., 2014). It can guide the differentiation of cells into chondrocytes in the periosteum, increase bone deposition and absorption, and plays an important role in the early stage of fracture healing (Yu et al., 2010). Consequently, BMP-2 can significantly promote the formation of mineralized nodules, osteogenic differentiation (Ball et al., 2019; Chen et al., 2019), mineralized bone length increase (Krishnan et al., 2001), bone healing (Sojo et al., 2005; Bessa et al., 2008), and limb development (Zhao C. et al., 2017). During the rapid ossification phase in the body, the mRNA expression of BMP-7 increases sharply and remains at a high level. The ability to induce Smad pathway expression is very significant, and it is inferred that BMP-7 may be a powerful promoter of bone development (Bosemark et al., 2015; Yan et al., 2018; Zhang et al., 2018; Li et al., 2019; Zhang L. et al., 2019). In clinical applications, in patients with acute open tibial fractures, rhBMP-2 and rhBMP-7 can promote nonunion healing and shorten the recovery time of patients (Caterini et al., 2016; Singh et al., 2016). The healing effect of BMP treatment is better than conventional treatment. Therefore, rhBMP-2 is a safe and effective method for autologous bone transplantation in the treatment of tibial fractures with large-scale traumatic bone loss (Jones et al., 2006).

In osteogenic cell lines, BMP-2, BMP-4, BMP-7, and BMP-9 effectively induce Smad-mediated signalling pathways. In addition, BMP-2, BMP-4, and BMP-9 induces Runx2 expression, but interestingly, BMP-6 and BMP-7 do not significantly induce Runx2 expression (Zhang L. et al., 2019). In recent years, research progress on BMPs has revealed that BMP-9 has the strongest ability to induce osteogenic differentiation of mesenchymal stem cells (MSCs) (Luu et al., 2007; Zhang L. et al., 2019). BMP-9 can effectively promote expression of the BMP/Smad signalling pathway, increase alkaline phosphatase (ALP) activity, induce the formation of mineralized nodules, and increase expression of osteogenic related genes, thereby promoting bone and joint regeneration (Wang et al., 2014; Chen et al., 2016; Yu et al., 2019). In addition, BMP-9 activates the Wnt/β-catenin signalling pathway, upregulates the protein level of β-catenin and promotes bone healing (Kusuyama et al., 2019; Wang H. et al., 2019). Table 2 lists the functions of BMPs in bone formation.

**TABLE 2:** Functions of BMPs in bone formation.

BMP(s)	Related GENE(s)	Biological function(s)	Reference(s)
BMP-2	Runx2, Dlx5, osteopontin (OPN), osteocalcin (OCN), and type I collagen	Promote the formation of mineralized nodule and osteogenic differentiation. Bone healing and limb development	Sojo et al. (2005), Bessa et al. (2008), Seo et al. (2014), Teotia et al. (2017), Ball et al. (2019), Chen et al. (2019)
BMP-4	Sox9, Acan, and type II collagen	Activating or promoting the release of other BMPs. Bone development, reconstruction and fracture healing	Fiedler et al. (2002), Jang et al. (2016)
BMP-5	Col2a1 and Sox9	Promote fracture and soft tissue healing	Guenther et al. (2015)
BMP-6	Col1a2, Runx2, and OPN	Significant osteogenic effect. Promote the healing of vertebral defect and segmental defect	Fischerauer et al. (2013); Pelled et al. (2016); Grgurevic et al. (2019); Toprak et al. (2021)
BMP-7	Runx2, ALP, OPN, and OCN	Induces the maturation of osteoblasts, and promotes the healing of fractures	Bosemark et al. (2015), Caterini et al. (2016), Singh et al. (2016), Kim et al. (2018), Yan et al. (2018)
BMP-9	ALP, Runx2, and type I collagen	The strongest ability to induce osteogenic differentiation	Luu et al. (2007), Wang X. et al. (2017), Zhang L. et al. (2019), Zhu et al. (2021)

### 2.3 Tendon/Ligament Healing

The human body needs not only the support of bones and articular cartilage but also a large number of muscles and ligaments to coordinate and pull the bones to complete various actions during daily activities. Therefore, tendons and ligaments are also important research directions in orthopaedics. However, since tendon and ligament cells are nonrenewable, only scar tissue can form, which will affect daily function. Therefore, how to fully restore tendons and ligaments is also a focus in the field of orthopaedics. In addition to cartilage formation and osteogenesis, BMPs also promote the differentiation and growth of tendons and ligaments, mainly BMP-12, BMP-13, BMP-14, and BMP-15 (Fu et al., 2003; Chuen et al., 2004; Wang et al., 2005; Haddad-Weber et al., 2010; Henn et al., 2010; Wang et al., 2018; Zhang L. et al., 2019).

BMPs have different transcriptional regulation effects on the important mediators of BMP signal transduction in BMSCs, and the most effective upregulators of tendon related gene are BMP-11, BMP-12, BMP-13, BMP-14 and BMP-15 (Zhang L. et al., 2019). Through morphological observation and molecular biological detection, MSCs have been found to be induced to differentiate into tendon cells by BMP-12 gene transfection, leading to type I collagen mRNA and protein expression but not type III collagen mRNA and protein expression (Wang et al., 2005). Cells transfected with BMP-12 were transplanted into rat tendon defects, and the results showed that type I/III collagen, scleraxis (SCX) and tenascin-C were all upregulated (Xu et al., 2018). Adenovirus-mediated implantation of BMP-13 into the supraspinatus tendon of rats revealed that BMP-13 upregulates the expression of type III collagen and fibronectin and increases biomechanical properties (Lamplot et al., 2014). Wang et al. (2018) studied the effect of BMP-14 on BMSC differentiation *in vitro*. BMP-14 upregulated sirtuin 1 (Sirt 1) expression at the mRNA and protein level, activated the JNK and Smad pathways, and significantly increased tendon markers, such as sclerotic and tonic regulatory proteins. BMP-12 and BMP-13 were expressed in MSCs and anterior cruciate ligament (ACL) fibroblasts via an adenoviral vector, and after 21 days of culture in a type I collagen hydrogel, both the MSCs and ACL fibroblasts differentiated into ligament cells (Haddad-Weber et al., 2010). Thus, combined application of BMPs also plays an important role in tendon and ligament healing. Table 3 lists the functions of BMPs in tendon/ligament healing.

**TABLE 3:** Functions of BMPs in tendon/ligament healing.

BMP(s)	Related GENE(s)	Function(s)	References
BMP-12	Type I collagen and SCX	Induced MSCs to differentiate into tendon cells	Wang et al. (2005)
BMP-12	Type I/III collagen, tenascin-C, and SCX	Promoted window defect regeneration	Xu et al. (2018)
BMP-13	Type III collagen, fibronectin	Improve rotator cuff tendon healing and reduce the incidence of rotator cuff	Lamplot et al. (2014)
BMP-14	Sclerotic and sirtuin1	Activate JNK and Smad pathways, induce the tendon differentiation of BMSCs	Wang et al. (2018)

### 2.4 Blood Vessel Formation

Angiogenesis is considered to be an essential key step in the process of bone regeneration because bone is highly vascularized. In the process of bone repair, newly formed blood vessels are very important for nutrition supply, macromolecular transport, cell aggregation and maintenance of the proper metabolic microenvironment. Since a defect site requires not only the continuous differentiation of MSCs but also blood vessels to provide nutrients, promotion of angiogenesis and sustained release of BMP are particularly important for promoting bone formation (Kempen et al., 2009). At present, vascular endothelial growth factor (VEGF) is considered to be one of the key regulators in angiogenesis (Peng et al., 2002). VEGF regulates the proliferation, vascularization and ossification of stem cells (Duan et al., 2016; Liu et al., 2020). In normal bone healing, vascular tissue development precedes bone formation. VEGF first stimulates angiogenesis, and BMP then promotes bone formation. Vascular tissue formation and bone formation are interdependent. BMP and VEGF have a regulatory coupling effect between osteogenesis and angiogenesis (Bouletreau et al., 2002; Barati et al., 2016; Zhong et al., 2016). The dual release of growth factors can promote the process of bone regeneration more effectively than either factor alone. Studies have shown that topical application of VEGF can promote bone repair in nonunion models, especially in the early stage of fracture healing (Percival and Richtsmeier, 2013; Li et al., 2021a; Li A. et al., 2021). Therefore, in order to promote bone regeneration more effectively, rapid early release of VEGF and sustained slow release of BMP-2 should be achieved, which is consistent with the law of bone growth and angiogenesis may be critical for bone tissue regeneration. VEGF and BMP-2 act together to significantly enhance osteogenesis and angiogenic differentiation (Barati et al., 2016). In the process of normal bone healing, high expression of VEGF occurs in the early stage, and high expression of BMPs occurs in the later stage. VEGF indirectly promotes bone formation by regulating formation of the vascular network. In addition, this interaction between BMP and VEGF has been confirmed in bone regeneration studies. By enhancing BMP- expression at the fracture site, the osteogenesis of osteoblasts can be directly stimulated, and the angiogenesis of endothelial cells can be promoted (Bouletreau et al., 2002; Kim et al., 2018).

### 2.5 BMP Derived Peptide

In general, BMP has some disadvantages such as high cost, ectopic bone formation, chemical instability, and immune response problems. BMP derived peptide has the advantages of small molecular weight, good chemical stability, flexible application and low economic cost, so it has higher application safety and biological effect (Beauvais et al., 2016; Li A. et al., 2021; Meng et al., 2021). BMP derived peptide can also activate osteogenic differentiation pathway and promote osteoblast differentiation, thus inducing differentiation and osteogenesis (Caron et al., 2021; Chao et al., 2021).

### 2.6 BMPs With Other Active Substances

Various biologically active substances and drug interventions have shown potential to promote fracture healing (Einhorn and Gerstenfeld, 2015). Bone healing relies not only on a single growth factor but also on the joint role of many biological components, such as dexamethasone (Dex), vitamin C, vitamin D, BMPs, TGF-β, VEGF, FGF, and insulin. At present, Dex is typically added to *in vitro* osteogenesis induction media. This is because Dex has a synergistic effect with BMP-2; when MSCs were cultured in basic medium supplemented with both BMP and Dex, osteoblast ALP activity was significantly increased, and osteoblast mRNA levels were increased (Rickard et al., 1994). Research shows that, the combination of BMP and FGF significantly enhanced osteogenesis than growth factor alone (Gromolak et al., 2020; Hu et al., 2021). Bmp-2 may stimulate extramedullary bone regeneration, and FGF-2 may stimulate extramedullary bone regeneration (Nosho et al., 2020). The combined use of BMP-2 and TGF-1 or TGF-3 showed stronger chondrogenic activity than BMP-2 alone (Yu et al., 2010; Sang et al., 2014). Early release of TGF-1 induces chondrogenesis, and BMP-2 promotes bone remodeling in a sustained release. TGF-β1 and BMP-2 showed good biocompatibility and bone formation ability in bone defects, which enhanced the chondrogenic ability of bone marrow mesenchymal stem cells (Dang et al., 2017; Li et al., 2021b). The combination of TGF-β3 and BMP-6 promoted chondrogenic differentiation of BMSCs in a synergistic manner (Taghavi et al., 2020).

## 3 Applications of BMPs in Bone Tissue Engineering

**FIGURE 2:**
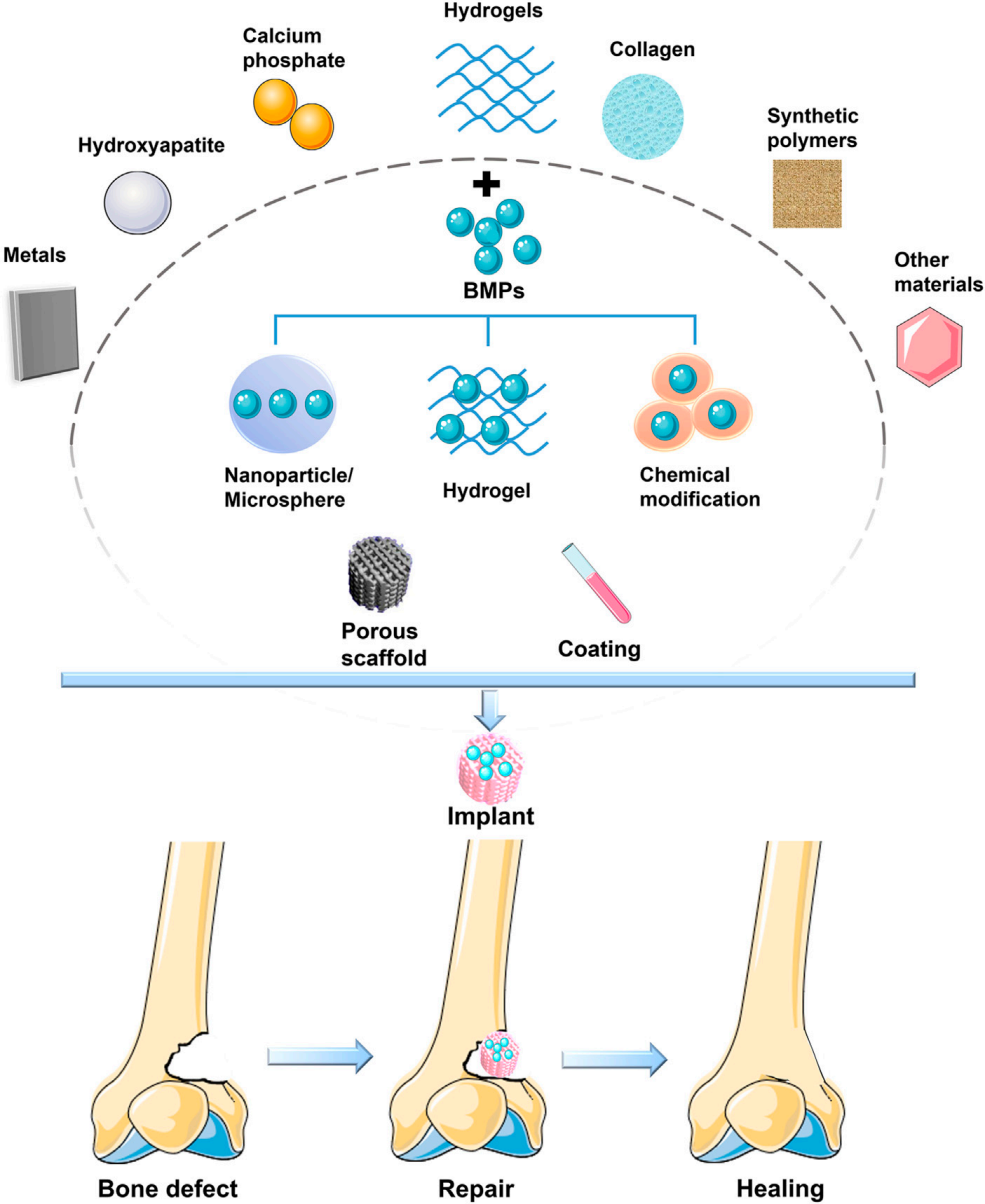
BMP is loaded on various materials to form a bioactive scaffold to promote bone defect healing.

BMPs have been shown to have strong cartilaginous, osteogenic and tendinous activity (Wang et al., 2005; Wang et al., 2018; Ball et al., 2019; Chen et al., 2019; Wang T. et al., 2019), and their applications in bone tissue engineering are promising. At present, most materials have poor retention of BMPs, leading to rapid clearance of BMPs from the site of implantation and thus to loss of the role of continuous stimulation of osteogenic differentiation of stem cells. BMPs can be slowly released in the body in several ways. The approach is to load BMPs into artificial implants, such as porous scaffolds, scaffold coatings or collagen combined with scaffolds (Figure 2) (Bouyer et al., 2016).

### 3.1 BMPs Combined With Scaffolds

In orthopaedic surgery, implants play a crucial role in the success of the operation, especially in bone defects that exceed a critical size and require matrix as a scaffold to guide bone regeneration. Therefore, the scaffold structure is of great significance for bone regeneration. Scaffolds are defined as special structures that allow cells to interact with the extracellular matrix and provide mechanical support for growing cells and tissues (Li et al., 2006; Ding et al., 2016; Wu S. et al., 2018). In the field of orthopaedics, the main role of the scaffold is bone conduction, and the scaffold combines with different types of cells and adjacent tissues to promote new bone formation (Kayabasi et al., 2013; Quade et al., 2020). Moreover, scaffolds can also be used as carriers for active molecules, such as cells and drugs, in the bone regeneration process to achieve better bone healing effects and good antibacterial properties (Ren et al., 2016; Huang et al., 2018).

#### 3.1.1 Loading and Releasing BMPs From Scaffolds

In the process of repairing bone defects, the addition of BMPs can play a significant role in promoting healing of bone defects. Although the osteogenic activity of growth factors is significant, most orthopaedic diseases require artificial implants to assist bone healing. The quality and quantity of bone formation at the defect site depend on the dose of BMPs (Teng et al., 2019). However, a large amount of BMPs released in a short period of time not only fails to effectively promote bone formation but may also cause side effects, such as heterotopic ossification and inhibition of bone formation. Thus, slow and continuous release of BMPs at the site of implantation has an important effect on bone regeneration (Barati et al., 2016; Seo et al., 2017). At present, artificial orthopaedic implants play a stable structural role at the defect site, while the role of slow and continuous release of bioactive substances needs to be further explored. To solve the problem of BMP release, it is very important to construct a sustained BMP release system using artificial implants. Therefore, the promotion of osteogenesis by BMPs loaded in various new materials has become a research hot spot (Tao et al., 2019; Chen et al., 2020).

#### 3.1.2 Connecting the Bone Defect Site and Promoting Bone Formation

The success of orthopaedic surgery depends on the degree of bonding between the implant and the surrounding bone. The higher the degree of osseointegration is, the higher the mechanical stability and the lower the possibility of loosening. Artificial implants can be implanted into the bone defect, connect the bone defect and combine with the surrounding bone tissue to achieve a stable mechanical structure and effectively stimulate bone formation (Rihn et al., 2009; Nam et al., 2017). However, implants have the problem of poor biocompatibility. If the implant is not specially treated, it will not combine well with the tissue in the human body, and it may cause bone nonhealing or bacterial infection, which ultimately leads to treatment failure. Modification of the implant through chemical or physical methods improves biocompatibility and helps the bone injury heal faster. In addition, bioactive molecules (such as BMPs) and drug delivery systems can be added to implants to further improve bone conductivity and antibacterial properties to promote bone healing (Liu et al., 2007; Han et al., 2014).

#### 3.1.3 Supporting the Growth of New Blood Vessels

The osteogenic induction ability of implants also depends on their ability to induce new blood vessel formation. The necessary conditions for the survival of cells and tissues growing in scaffolds are supplied by blood vessels. The injury site is induced to form a complex vascular network in the scaffold, which can provide abundant bone progenitor cells at the bone defect site, stimulate the migration and differentiation of osteoblasts, lead to increased bone deposition, and thus promote bone healing (Kempen et al., 2009; Kim et al., 2018).

### 3.2 Loading Strategies of BMPs

In composite scaffold systems, bioactive proteins are usually adsorbed to the surface of porous material or encased in pores (Injamuri et al., 2020). As a biologically active substance, BMP may be inactivated or released suddenly during the process of loading into the composite scaffold. In addition, after loading BMP on the surface of the scaffold, the interaction between the BMP and the surface of the biological material may be destroyed due to the interaction between the material and the organism. These factors may cause BMP to fail to achieve the desired sustained release effect in many composite scaffolds (Rajabnejadkeleshteri et al., 2021). Therefore, the current research should focus on the development of stable composite scaffolds to achieve stable release of BMP.

#### 3.2.1 Nanoparticle/Microsphere

Microspheres/nanoparticles can be directly used as carriers to deliver growth factors to bone defects. Growth factors were encapsulated in an intermediate delivery tool consisting of microspheres to provide a protective barrier that would not affect their biological activity during scaffold fabrication. In addition, the slow-release ability of microspheres enables BMP to be released stably and sustainably at the target site, which can effectively promote bone tissue repair. At present, microspheres are mainly divided into natural polymer microspheres and synthetic polymer microspheres. The microspheres are wrapped with BMP through microcapsules, processed into composite scaffolds, and implanted into bone defects to achieve the purpose of continuous induction of bone regeneration (Bai et al., 2020; Injamuri et al., 2020; Kong et al., 2020; Koons et al., 2021; Rajabnejadkeleshteri et al., 2021).

#### 3.2.2 Hydrogel

Hydrogels have been widely studied for BMP delivery due to their injectable, easy chemical modification, degradability, and permeability of macromolecules. While the hydrogel continues to degrade, BMP will also be continuously released. At the same time, because the hydrogel has better permeability, tissues and cells can also better absorb the BMP in the hydrogel (Larochette et al., 2020; Datta et al., 2021; Lee et al., 2021).The clinically approved delivery method of BMP is usually adsorbed on a collagen sponge and then implanted into the bone defect. Therefore, in orthopaedic applications, the hydrogel can be filled alone, used as a coating for implants, or used to fill porous materials to provide a greater degree of coverage in the gap between the implant and the bone, so as to better stimulate bone growth.

#### 3.2.3 Chemical Modification

Due to the biological activity characteristics of BMP, it can form a more stable combination of some chemical substances through chemical bonds, thus forming a bioactive delivery system. Heparin, a natural glycosaminoglycan, is a molecule that composes the extracellular matrix (ECM), which participates in the binding and isolation of growth factors in the cell microenvironment. Heparin has a strong affinity for BMP and has the benefit of enhancing the biological activity of BMP. Heparin-binding peptides are used in drug delivery systems and show better BMP binding and controlled delivery *in vivo*. Such as heparin methacrylamide microparticles (HMPs), HMPs can be incorporated into the hydrogel to adjust the rate of release of BMP-2 from the scaffold (Hettiaratchi et al., 2020; Subbiah et al., 2020). Current research shows that compared with collagen sponges, HMPs drug delivery system has significantly increased bone formation, reduced heterotopic ossification, and regular bone formation (Vantucci et al., 2021). The heparin delivery system provides a better choice for improving the clinical use of BMP-carrying methods.

### 3.3 Structural Characteristics of BMP- Loaded Scaffolds

#### 3.3.1 Porous Structure

**FIGURE 3:**
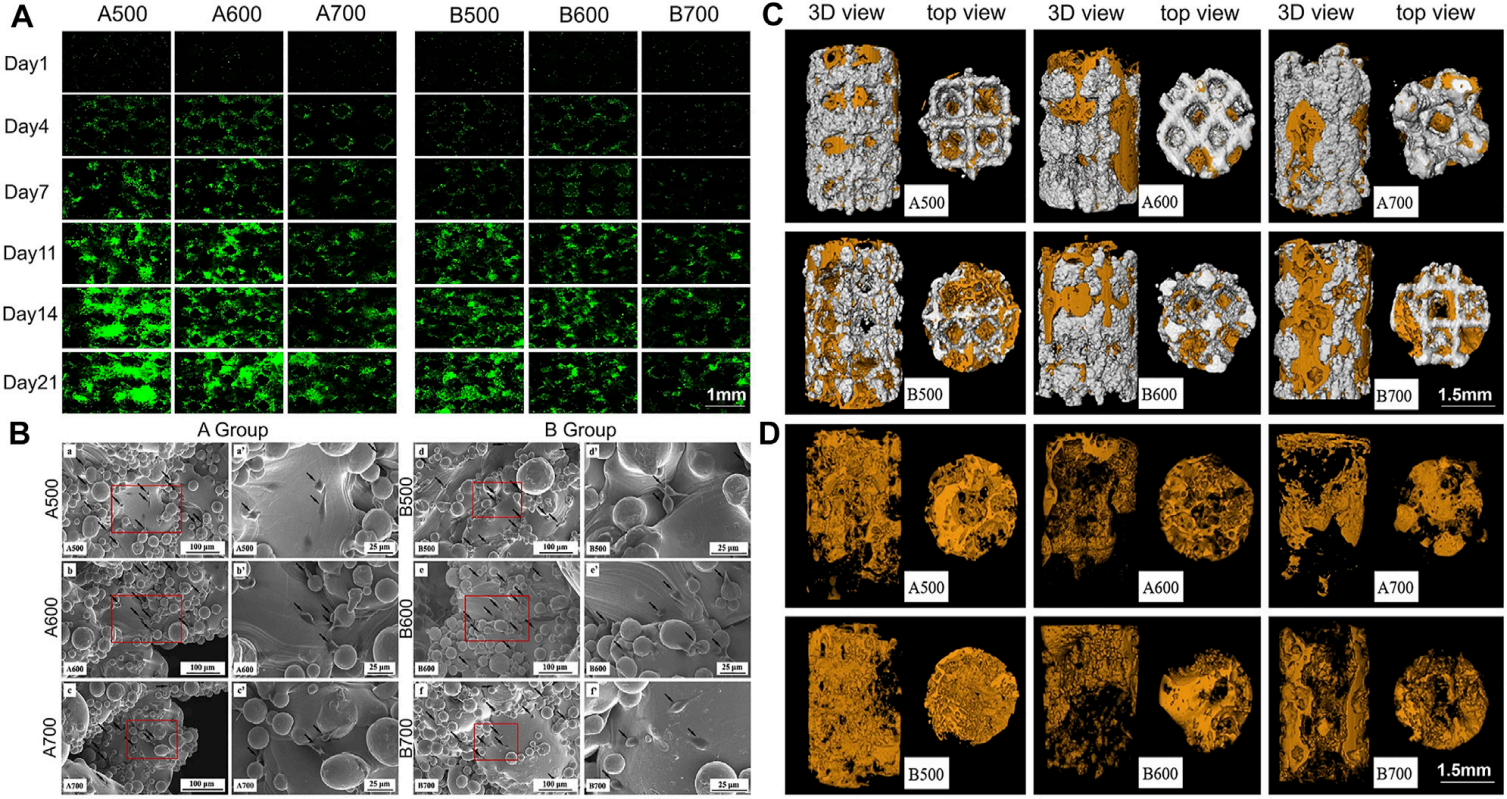
**(A)** Fluorescent images showing cell proliferation on the porous scaffolds. **(B)** Scanning electron microscopy (SEM) observation of rat BMSCs. Black arrows point to cells. **(C)** Micro-CT analysis of new bone formation. Reconstruction images of porous implants; **(D)** Reconstruction images of bone formation. Orange area: new bone formation. Reprinted with permission from (Chen et al., 2020).

Biomaterial scaffolds with well-connected porous structures play an important role in bone tissue engineering. The porous structure is conducive to the adhesion, proliferation and differentiation of mesenchymal stem cells, allowing the cells to interact with the extracellular matrix to provide mechanical support for growing cells and tissues (Ding et al., 2016; Li et al., 2018; Wu S. et al., 2018). On the one hand, the presence of a porous structure allows cells to quickly diffuse into the scaffold; on the other hand, it provides a higher interface bonding area for vascularization and bone growth, which can better promote immobilization of implants and bones. More importantly, the scaffold should have interconnected pores and high porosity so that the infiltration and proliferation of cells, the growth of blood vessels, the diffusion of nutrients and the elimination of waste will achieve better results. By adjusting the pore size and porosity, an implant with the best density, strength and mechanical compatibility can be obtained, which can effectively prevent bone necrosis and bone deformity around the implant and effectively improve the success rate of orthopaedic surgery (Figure 3) (Kayabasi et al., 2013; Han et al., 2014; Nam et al., 2017; Teng et al., 2019; Zhang X. et al., 2019; Chen et al., 2020; Quade et al., 2020; Zhu et al., 2021).

#### 3.3.2 Coating

Coating the surface of an implant reduces the gap around the implant, avoids direct contact between the implant and bone and reduces osteolysis caused by implant movement and wear at the implant site, thereby increasing opportunities for stable interface formation (Kayabasi et al., 2013; Ishack et al., 2017; Behrendt et al., 2020). Moreover, coatings can be loaded with BMPs to control their slow and long-term release to reduce the side effects of short-term overrelease of BMPs and enhance the fusion of implant bone with the surrounding bone. Therefore, a stable coating can greatly increase the success rate of implantation and osseointegration (Kayabasi et al., 2013; Shen et al., 2016; Wu S. et al., 2018; Zhu et al., 2021). At present, coating technology is developing rapidly, such as dip coating, bionic coating, sol-gel, electrophoretic deposition, vapour deposition, laser processing, ion spraying and 3D printing (Teng et al., 2019; Li et al., 2021a; Koons et al., 2021).

Incorporation of BMPs into a bionic coating allows high pharmacodynamic activity at a low pharmacological level and maintenance of this activity for a long period of time. Improved implants with coatings have good bone induction and bone conduction ability, which can not only promote cell adhesion and proliferation but also effectively promote osteogenic differentiation and improve biological activity (Wu J. et al., 2018; Teng et al., 2019). The maintenance of this osteogenic activity has important clinical significance for the osseointegration of the implant (Liu et al., 2005).

### 3.4 Loading of BMPs in Different Biomaterials

#### 3.4.1 Metals Based Composite Scaffolding System

Among implant metal materials, titanium alloys are the most widely used orthopaedic implant materials due to their outstanding characteristics, such as high strength, high corrosion resistance, and biological inertness. However, due to the naturally inert nature of titanium alloys, they do not have sufficient biological activity and cannot be well combined with bone tissue; thus, there may be a gap between the bone and the implant, leading to unstable healing and resulting in failure of bone healing (Sul et al., 2002; Geetha et al., 2009; Li et al., 2021a). The porous structure of titanium alloy is constructed by 3D printing and other technologies, and surface modification of titanium alloy is carried out using various methods, such as electrodeposition, so that biologically active molecules (such as BMP) can be loaded on the surface coating of titanium alloy to achieve slow and continuous release. Therefore, bone formation at the bone defect site is continuously stimulated, the degree of bonding between the implant and the bone interface is enhanced, and the bone healing interface is more stable (Tao et al., 2019; Teng et al., 2019; Chen et al., 2020; Zhu et al., 2021).

**FIGURE 4:**
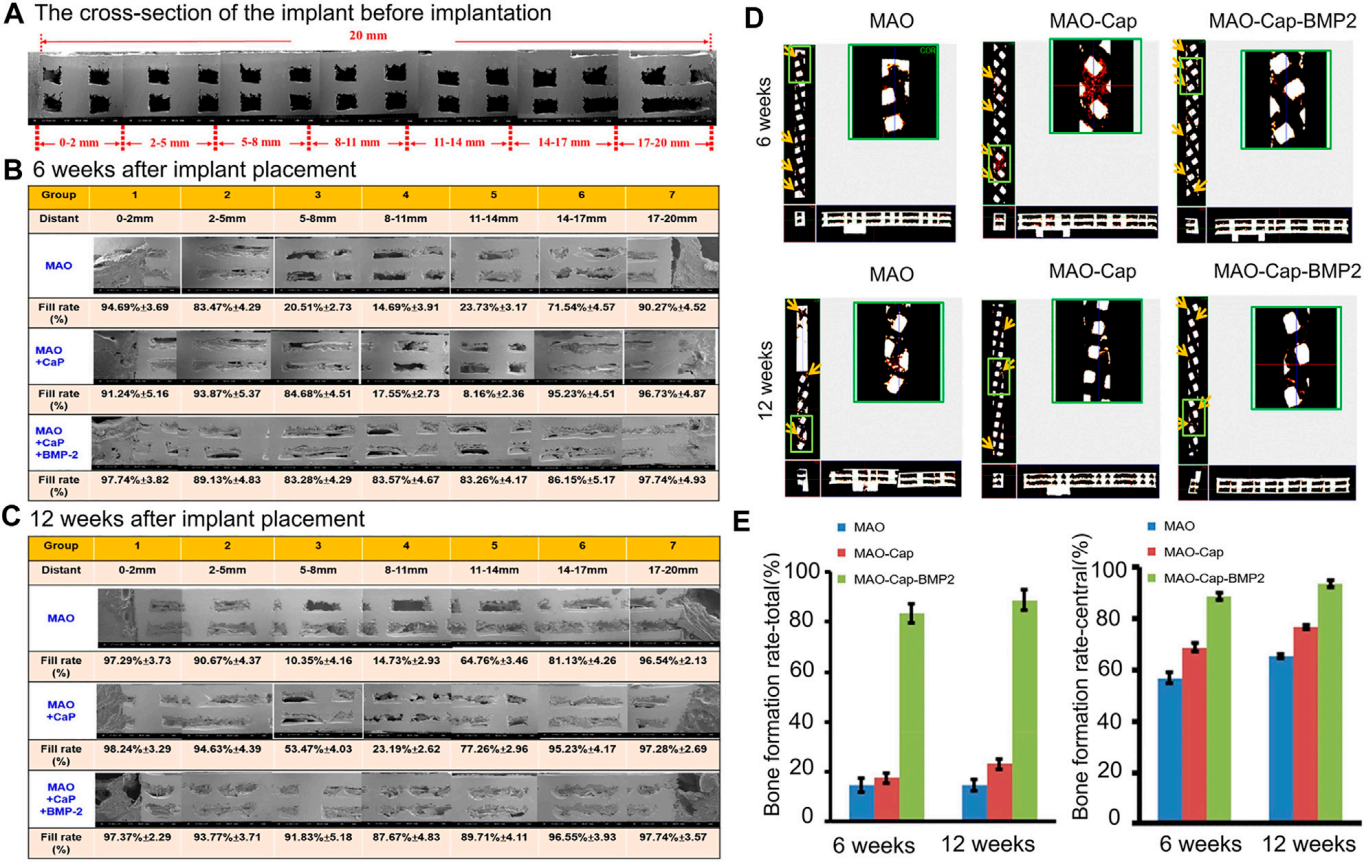
**(A)** SEM image showing the cross-section of the implant before implantation. **(B,C)** are cross-sectional scanning electron microscope images 6 and 12 weeks after implant placement. **(D)** The blood vessel growth into the implant at 6 and 12 weeks observed by Micro CT. Red dots: newly formed blood vessels. **(E)** Using ImageJ software to estimate the bone filling rate of the cross-sectional scanning electron microscope image. Reprinted with permission from (Teng et al., 2019).

Titanium alloys can be optimized not only by surface modification but also by other processing techniques, including a variety of materials, porous structures, nanotechnology applications, and 3D printing technology applications. Ti6AI4V is the most widely used titanium alloy material (Chen et al., 2020). Current research shows that the pores of titanium alloy scaffolds are 400–600 μm, and the porosity is 60–80%, which can promote cell proliferation, osteogenic differentiation and bone growth and can improve the degree of bone–scaffold binding (Han et al., 2014; Taniguchi et al., 2016; Zhang T. et al., 2020). In addition, surface modification of titanium alloy can achieve faster osseointegration of bone and implants in the early stage and can achieve certain antibacterial ability to facilitate stable chemical bonds between implants and bone tissue. Increasing research has been carried out on the surface modification of titanium alloys. Through various physical and chemical methods, materials with good biocompatibility can be stably combined on the implant surface, such as hydrogels, polymers, hydroxyapatite, calcium phosphate, and bisphosphonate (Nie and Wang, 2007; Hu et al., 2012; Han et al., 2014). Basic fibroblast growth factor (bFGF) and BMP-2 incorporated in a polydopamine (PDA) coating on a titanium surface led to an obvious improvement in surface hydrophilicity, efficient growth factor adsorption and moderate sustained release of the modified titanium matrix (Wu et al., 2020). In one study, BMP-2, calcium, and phosphorus (Ca/P) coatings were codeposited with 3D-printed porous titanium alloy implants and treated via microarc oxidation (MAO), achieving continuous release of BMP-2 over a period of 35 days. The continuous release of BMP-2 effectively improved ALP activity and *in vitro* mineralization and promoted new bone formation *in vivo*. The BMP-2 and Ca/P coating on titanium alloy produced better osseointegration and further promoted bone formation compared with pure titanium alloy or Ca/P-coated titanium alloy (Figure 4) (Teng et al., 2019). Titanium dioxide (TiO2) nanotubes are also one of the more widely used materials. The advantage of this material is that it is receptive to loading of drugs and growth factors. Titanium dioxide was used as a carrier of BMP-2, sodium alginate, gentamicin and chitosan (CHI), and this modification improved the biocompatibility and enhanced the antibacterial ability and bone formation ability of the composite (Tao et al., 2019).

Modified titanium alloy is more suitable for implantation in multiple parts of the body, has better biocompatibility with the human body, promotes bone healing and angiogenesis, reduces the bone healing failure rate, and plays a role in healing of bone defects caused by trauma.

#### 3.4.2 Inorganic Salts Based Composite Scaffolding System

**FIGURE 5:**
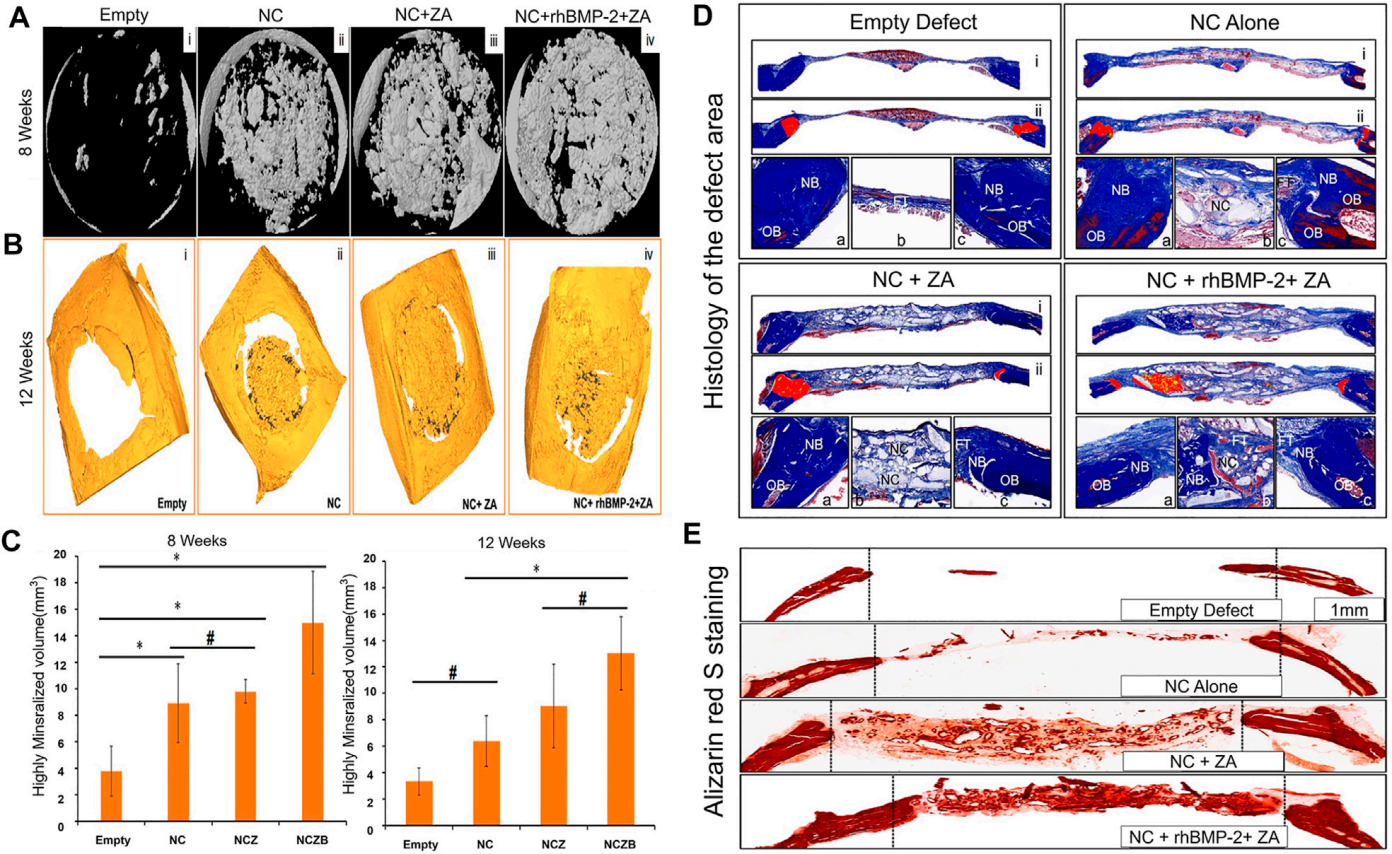
**(A)** Micro-CT shows a three-dimensional model image of the defect at 8 weeks. **(B)** Micro-CT shows the image of the defect at 12 weeks. The defect is highly mineralized and integrated with bone material. **(C)** Morphological analysis of the defect site based on micro-CT, showing the high mineralization of the defect site at 8 and 12 weeks (*p < 0.05; #no statistical differences between the groups). **(D)** Histology of the defect area after 12 weeks. Each panel is divided into three sub-panels, where panel **(i)** (40 ×) represents an overall view, Panel **(ii)** represents a pseudo-color image, where “red” represents new bone (40 ×) from the original defect site. The bottommost panel shows a high-magnification image (100 ×) of the left side of the defect **(a)**, the area in the middle of the defect **(b)** and the right side of the defect **(c)**. OB stands for old bone, NB stands for new bone, FT stands for fiber/connective tissue, and NC stands for nano-cement residue. **(E)** Alizarin red S staining results are similar to the above results. The NC + rhBMP-2 + Za group had the most alizarin-positive (calcium-specific) mineralized tissues, followed by the NC + Za group. The number of alizarin-positive tissues in the NC group alone was less than in the first two groups. After 12 weeks, alizarin red S staining in the non-decalcified bone defect area showed a typical difference in calcium deposition between the groups (40 ×). Reprinted with permission from (Teotia et al., 2017).

Hydroxyapatite (HA) is the main inorganic component of human and animal bones. HA stimulates or induces bone proliferation, which can promote defective bone tissue repair. Therefore, it has excellent biocompatibility and is applied in bone tissue engineering. HA is widely used and has a good clinical effect (Kim et al., 2006; Ko et al., 2013; Bosemark et al., 2015; Wu S. et al., 2018; Li A. et al., 2021). The combined application of HA, autologous BMSCs, BMPs and autologous bone grafting (ABG) has been proven to be safe, with good stability and bone regeneration characteristics, and can be an option for treatment of severe bone defects (Teotia et al., 2017; Dilogo et al., 2019). HA can not only be used directly as a scaffold for bone defects but can also be mixed with other materials to form a composite scaffold or fixed to an implant surface, such as a titanium alloy implant, *via* coating (Nie and Wang, 2007; Ding et al., 2016). In addition, a nanohydroxyapatite (NHA) coating has a strong adsorption capacity and can be used as a suitable coating for BMP, providing a rich active site for cell attachment, which is more conducive to stable combination of the bone and the implant (Shen et al., 2016; Deng et al., 2017; Wu S. et al., 2018; Li A. et al., 2021). NHA and calcium sulfate bone substitute (NC) were used as carriers of rhBMP-2 and zoledronic acid (Za) to repair skull defects in rats, and the results showed that it could significantly promote bone regeneration (Figure 5) (Teotia et al., 2017).

Calcium phosphate can improve the bioactivity, bioabsorbability, bone conductivity and osteoinductivity of bioceramics. Biphasic calcium phosphate (BCP) and tricalcium phosphate (TCP) have become research hotspots in the field of bioceramics. BCP and TCP scaffolds have a high degree of biocompatibility and can support the attachment, proliferation and differentiation of osteoblasts, thereby promoting the formation of bone tissues. Thus, increasing research has been conducted on these materials (Bosemark et al., 2015; Ishack et al., 2017; Quade et al., 2020; Chao et al., 2021). Although calcium phosphate shows a chemical composition similar to that of inorganic bone, its osteogenic performance is restricted by its low degradation rate and poor bone induction. Therefore, it is necessary to modify the material to obtain a better performance. Mixing of calcium phosphate and HA to make a scaffold has received increasing attention. However, the delayed or rapid biodegradation rate of HA may interfere with the rate of new bone formation. The main advantage of calcium phosphate is that it has higher chemical stability, and a better biodegradation rate can improve the bone repair effect (Lee et al., 2016). Combining HA and calcium phosphate makes a composite implant more similar to bone minerals, with better biological activity, biodegradability and mechanical properties, which can achieve better therapeutic effects (Kim et al., 2006; Ko et al., 2013).

**FIGURE 6:**
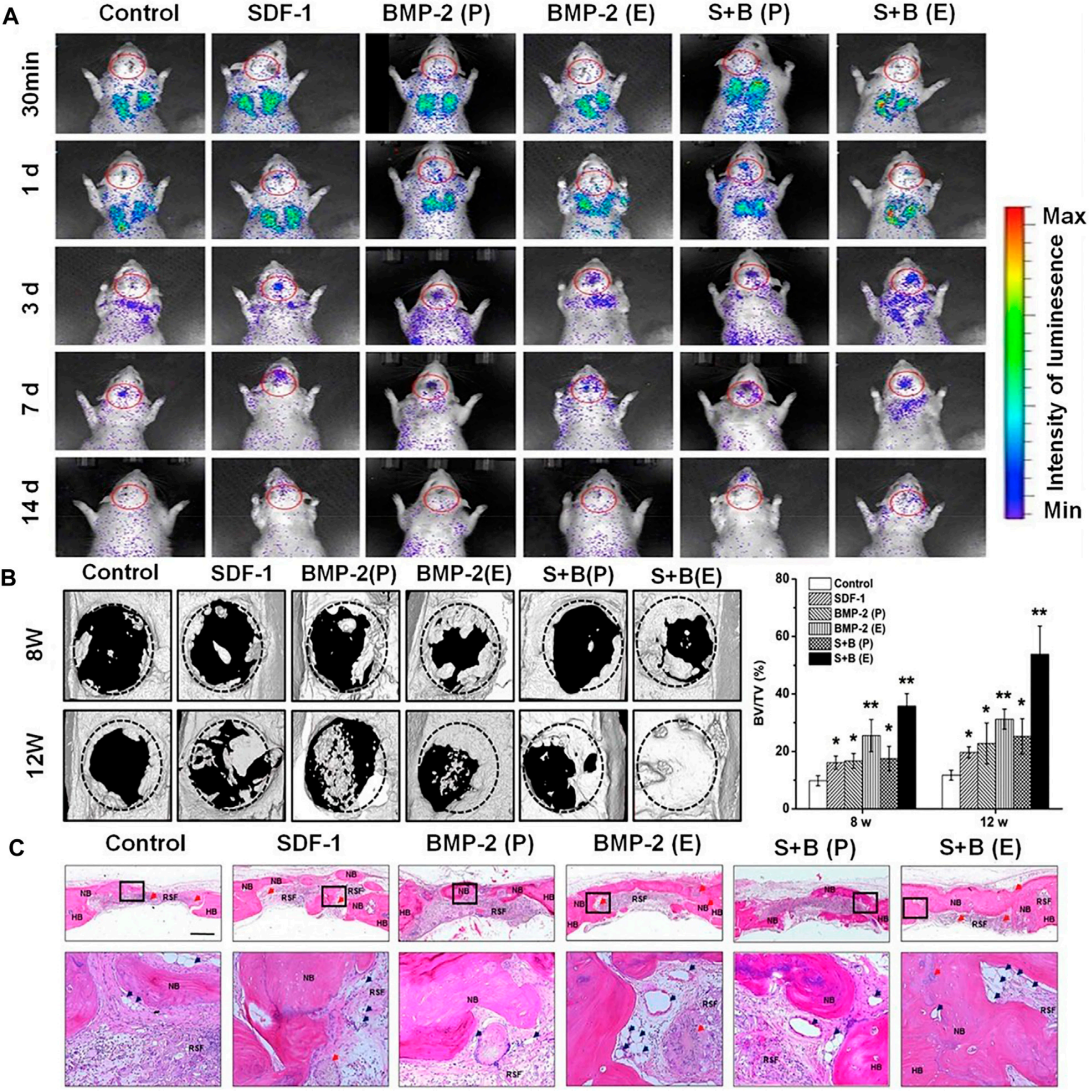
**(A)** BMSC migration to the defect cranial site. Rats are shown at 30 min, 1, 3, 7 and 14 d post-injection; data indicate the systemic cell distribution of reporter cells. Red circles indicate the ROI. **(B)** CT results of critical size defect specimens and bone volume distribution. The circular plate in **(B)** represents the original skull defect. The white area above the circular plate and the light area inside the plate represent the newly formed bone. **(C)** Repair the skull cross-sectional structure (25 ×) with a stent 12 weeks after implantation. Ruler = 1 mm. After H & E staining, bone-like structures were detected in each group. The second line represents a higher magnification image (200 ×) of the corresponding box in the first line in **(C)**. NB, HB and RSF represent new bone, host bone and residual silk fibroin, respectively. The red arrow shows the remaining microspheres. Blue arrows indicate newly formed blood vessels. BMP-2 (P): the scaffold material that physically adsorbs BMP-2, BMP-2 (E): SF microsphere coated BMP-2 scaffold, S + B (P): Scaffold material that physically adsorbs SDF-1 and BMP-2, S + B (E): The physical adsorption scaffold of SDF-1 and BMP-2 in SF microspheres. Reprinted with permission from (Shen et al., 2016).

At present, due to the natural osteogenic properties of HA, more and more studies have been conducted on HA composite scaffolds. In one study, Bosemark et al. (2015) made tricalcium phosphate hydroxyapatite composite scaffolds carrying BMP-7 to study the repair of femoral defects in rats. Compared with the use of scaffolds or drugs alone, the callus characteristics of bone defects were significantly improved. In another study, the composite scaffold was made according to the ratio of 15% HA:85% β-TCP, and it has the advantages of good bone conduction and bone integration (Ishack et al., 2017). Wu S. et al. (2018) made a NHA/collagen/poly (L-lactide) composite scaffold loaded with rhBMP-2 to treat bone defects that had a positive effect on human MSCs implantation, proliferation and osteogenic differentiation. Shen, XF and others have made composite scaffolds with silk fibroin (SF) and NHA, in which BMP-2 was loaded into SF microspheres, BMP-2 can be sustained and slowly released for up to 3 weeks. Therefore, the composite scaffold can continuously and slowly release BMPs and significantly promote osteogenic differentiation of BMSCs (Figure 6) (Shen et al., 2016). In summary, HA composite systems have broad application prospects in bone tissue engineering.

#### 3.4.3 Hydrogels Based Composite Scaffolding System

Hydrogels are extremely hydrophilic gels with a three-dimensional network structure that swell rapidly in water and can absorb a large volume of water without dissolving. The hydrogel is neither a complete solid nor a complete liquid. Under certain conditions, hydrogels can maintain a certain shape and volume, and a solute can diffuse from or penetrate the hydrogel (Li et al., 2006; Rihn et al., 2009; Jin et al., 2010; Kayabasi et al., 2013; Zhang X. et al., 2019). Therefore, in orthopaedic applications, a hydrogel can be filled separately, used as a coating for implants, or be used to fill porous materials to provide a greater degree of coverage in the gap between the implant and bone to stimulate bone growth and avoid loosening of the implant (Hu et al., 2012; Kayabasi et al., 2013; Behrendt et al., 2020). Moreover, studies have shown that hydrogels can also transfer cells, which is conducive to the growth of cells and can continuously stimulate the potential of BMSC differentiation (Ben-David et al., 2011; Hu et al., 2021). Therefore, hydrogels have been widely used in orthopaedic treatments as an implant material, coating or filling in the pore structure of a material, with the functions of controlled release and sustained release. Collagen, fibrin, CHI, agarose, hyaluronic acid, silk and sodium alginate can be configured into hydrogels to carry BMP for more convenient and effective application to bone injury sites (Ben-David et al., 2011; Kayabasi et al., 2013). In clinical applications, combined application of hydrogels and BMPs has achieved good results (Kolambkar et al., 2011; Nam et al., 2017; Jain et al., 2019; Xu et al., 2019).

Collagen is one of the most abundant ECM proteins and the most commonly used synthetic hydrogel material. Due to its good biocompatibility, nutrition, reparability, moisture and affinity, collagen is widely used in biomedical materials (Rihn et al., 2009; Nam et al., 2017). However, due to the instability and rapid degradation of collagen, artificial collagen implants cannot maintain their structural integrity for a long time. Currently, collagen can be modified to prolong durability and mechanical strength and can be combined with other materials so that implants can achieve slow release of loaded BMPs (Li et al., 2006; Kayabasi et al., 2013). By optimizing the physical and chemical properties of collagen, the best environment for bone tissue development can be provided, greatly improving the efficiency of bone regeneration (Li et al., 2021c). To date, rhBMP-2 and rhBMP-7 has been approved by the FDA for use in combination with collagen sponges in the treatment of clinical diseases and has achieved good efficacy (Kolambkar et al., 2011; Jain et al., 2019; Xu et al., 2019).

In one study, Acellular Bioactive affinity-binding Alginate hydrogel was designed to slowly release a chondral and osteogenic inducer (TGF-β1 and BMP-4, respectively). Hydrogel was injected into the osteochondral defect of the medial femoral condyle of miniature pigs. After 6 months, histological evaluation showed that the articular cartilage layer was effectively reconstructed with the major features of hyaluronic cartilage, such as proteoglycans and type II collagen deposition. The results showed that treatment with an affinity-binding alginate saline gel containing cell-free injectable growth factors was effective in repairing the tissue and had the main characteristics of hyaline cartilage (Ruvinov et al., 2019). CHI has a wide range of applications in bone tissue engineering and antibacterial activity. CHI scaffolds and BMP-6-transfected rat BMSCs were used to treat bone defects and promote cartilage formation (Kayabasi et al., 2013). A bioactive multilayer structure of gelatine/CHI containing BMP-2 and fibronectin was constructed on the surface of Ti6Al4V, which was beneficial to osteogenic differentiation and integration of implant and bone (Hu et al., 2012). Current research shows that composite hydrogels have more significant osteoinductive activity and better development prospects when applied together with other materials (Kolambkar et al., 2011; Dhivya et al., 2015; Ghavimi et al., 2019; Ruvinov et al., 2019).

#### 3.4.4 Synthetic Polymers Based Composite Scaffolding System

Synthetic polymers have been widely used in bone tissue engineering because of their excellent biocompatibility and biodegradability, and they can be combined with various materials to make composite scaffolds. In recent years, synthetic polymers have been widely used in aerospace, medical, dental, automotive, and other related fields. Especially in the field of orthopaedics, because synthetic polymers have excellent biocompatibility and good mechanical properties, they are easily produced and have certain bone-like properties, which help to extend the life of the implant (Toth et al., 2006; Wu J. et al., 2018). In terms of orthopaedic implant stents for treating disease, synthetic polymers are suitable for the manufacture of implants with high quality and high precision requirements, such as implants for joint, spine, skull, and maxillofacial surgery and other operations (Abu Bakar et al., 2003; Meisel et al., 2008; Vaidya et al., 2008). With the development of 3D printing technology, synthetic polymers can be used to manufacture implants that are highly matched to the patient according to the situation at the injury site in the patient, which can greatly improve the success rate of surgery and the long-term prognosis (Wu et al., 2015).

Polyether ether ketone (PEEK) is a thermoplastic organic polymer with excellent strength and stability, bone-like stiffness, high-temperature durability and wear resistance (Han et al., 2014). In clinical applications, patients with degenerative lumbar disease were treated with pedicle screws and PEEK cages for dorsal fixation and then treated with rhBMP-2. At 6 months of controlled evaluation, all cases met the criteria for spinal fusion (Meisel et al., 2008). PEEK can be mixed with titanium alloy, HA, TCP and other materials to improve mechanical properties and biocompatibility and achieve better therapeutic effects (Tan et al., 2003; von Wilmowsky et al., 2008; Han et al., 2014). PEEK-Ti6AI4V has better osseointegration capacity (Zhang W. et al., 2020). Nano-TiO2 can improve the biocompatibility and bone conductivity of PEEK (Han et al., 2014). Surface modification of microporous PEEK with BMP-2-loaded phosphorylated gelatine can significantly promote cell adhesion and proliferation, effectively promote osteogenic differentiation and improve biological activity (Wu J. et al., 2018). Therefore, the use of PEEK in orthopaedics has received extensive attention.

**FIGURE 7:**
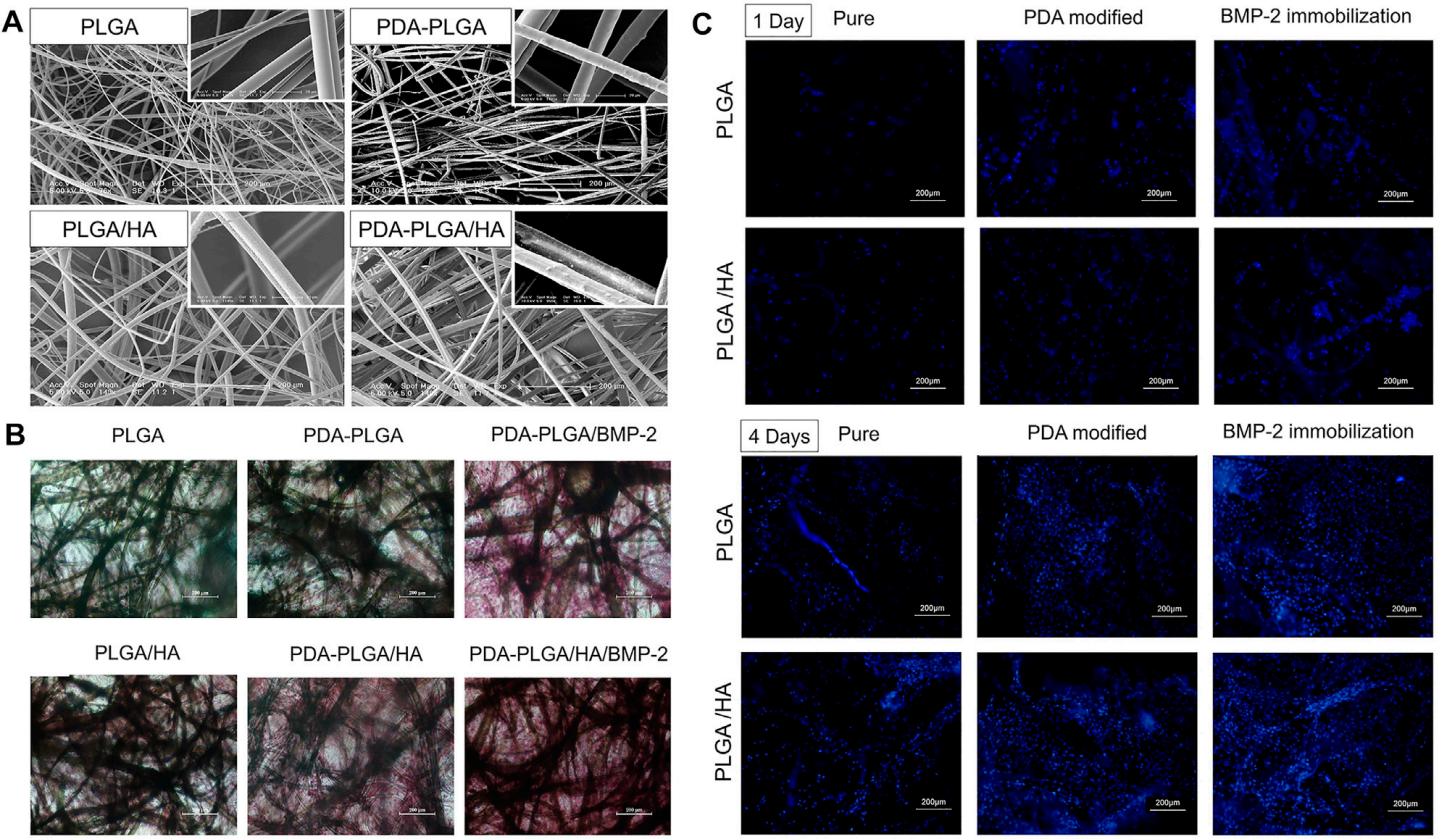
**(A)** SEM micro-photographs of PLGA, PDA-PLGA, PLGA/HA, and PDA-PLGA/HA scaffolds. **(B)** Alizarin Red staining of MC3T3-E1 cells cultured on PLGA, PLGA/HA, PDA-PLGA, PDA-PLGA/HA, PDA-PLGA/BMP-2, PDA-PLGA/HA/BMP-2 on day 21. **(C)** Fluorescence staining of MC3T3-E1 cells cultured on the different scaffolds for 1–4 days. Reprinted with permission from (Zhao X. et al., 2017).

Polylactic acid (PLA) is a new type of biodegradable material produced using raw starch materials obtained from renewable plant resources and subsequent synthesis of polylactic acid of a certain molecular weight through chemical synthesis methods (Zhang X. et al., 2019). Poly (lactic-co-glycolic acid) (PLGA) is polymerized from lactic acid and glycolic acid according to a certain ratio (Zhao X. et al., 2017). It is a degradable organic polymer compound. The degradation rate of PLGA is faster than that of PLA. Both PLA and PLGA have good biocompatibility, degradability, and plasticity; low cost; and good medical uses. Moreover, both PLA and PLGA have been approved by the FDA for clinical treatment. Therefore, PLA and PLGA have become widely applied in recent years (Zhang X. et al., 2019). PLA and PLGA can also be mixed with other materials. PLA/PLGA composite scaffolds have good stem cell loading properties and can induce cell-cell synergy, promote bone regeneration, and achieve better therapeutic effects (Laurencin et al., 2001; Zhao X. et al., 2017; Wu S. et al., 2018; Zhang X. et al., 2019). Zhang X. et al. (2019) used 3D printing technology to print a cylindrical PLA scaffold, which was surface-modified with dopamine (DA) and equipped with BMP-2. Then, the scaffold was implanted into a rat skull defect model. At 8 weeks after surgery, bone regeneration occurred in the skull defects of rats, and the fibrous bone tended to connect to form continuous bone tissue. Wu S. et al. (2018) made a NHA/collagen/PLA scaffold loaded with rhBMP-2, and the scaffold significantly increased phosphate content, mineral production, ALP activity and osteogenic biomarkers (OCN and Runx2). This complex had a positive effect on the proliferation and osteogenic differentiation of human MSCs. A PLGA-HA scaffold loaded with BMP-2 showed a significant promoting effect on cell adhesion and proliferation. In addition, genes related to alkaline phosphatase activity, calcium deposition and osteogenesis were highly expressed in cells (Figure 7) (Zhao X. et al., 2017). PLGA/HA composite scaffolds have good application prospects for bone regeneration (Laurencin et al., 2001; Nie and Wang, 2007). Importantly, the biological and mechanical properties of single-material scaffolds often fail to achieve the desired results. Compared with single-material scaffolds, composite material scaffolds have improved properties, show a more obvious effect on promoting bone defect healing, and can stimulate bone formation continuously, long-term and effectively (Bosemark et al., 2015; Ding et al., 2016). Table 4 lists application of BMPs in materials.

**TABLE 4:** The application of BMPs in materials.

BMP	Material(s)	Modified	Function(s)	References
rhBMP-2	Ti6Al4V	3D printing porous structure, pore size:400–600 μm, porosity:60–80%	Improved the degree of bone-scaffold bonding	Zhang T. et al. (2020)
BMP-2	Ti6Al4V	Porous structure, pore size:600 μm, prepared by a combination of MAO, calcium-phosphorus co-precipitation and electrodeposition BMP-2 coating technology (MAO-Ca/P-BMP2)	Bone induction and bone conduction capabilities, enhances the growth of cells, enables the formation of blood vessels in the implant and has a better osteogenic effect	Teng et al. (2019)
BMP-2	TNTs	Through layer-by-layer assembly technique, the sodium alginate and gentamicin and CHI were constructed on BMP 2 loaded TNTs substrate	Enhanced antibacterial ability and bone formation ability	Tao et al. (2019)
BMP-2	HA	Combination of autologous BMSCs and ABG	Enhanced stability and bone regeneration characteristics	Teotia et al. (2017), Dilogo et al. (2019)
BMP-2	NHA	NHA coating	Providing a rich active site for cell attachment, which is more conducive to the stable combination of bone and implant	Deng et al. (2017)
BMP-7	TCP	Carried BMP-7 and bisphosphonates	Improved bone defects, promoted bone healing	Bosemark et al. (2015)
	HA			
BMP-2	HA	Composite scaffold, ratio: 15% HA: 85% β-TCP	Improve bone conduction and bone integration	Ishack et al. (2017)
	β-TCP			
rhBMP-2	NHA	Composite scaffold	Positive effect on human MSCs implantation, proliferation and osteogenic differentiation	Wu S. et al. (2018)
	Collagen			
	PLA			
BMP-2	SF	Composite scaffold	Continuously and slowly release growth factors and significantly promote the osteogenic differentiation of BMSCs	Shen et al. (2016)
	NHA	SF microspheres stromal cell-derived factor-1 (SDF-1) is bound to the scaffold		
BMP-2	Ti6Al4V	Layer-by-layer assembly technology, construct a bioactive multilayer structure of gelatin/CHI containing BMP-2 and fibronectin on the surface of Ti6Al4V	Beneficial to osteogenic differentiation and integration of implant and bone	Hu et al. (2012)
	CHI			
BMP-6	CHI	CHI scaffolds and BMP-6 transfected rat BMSCs	Promote bone formation and cartilage formation	Kayabasi et al. (2013)
rhBMP-2	PEEK	Pedicle screw and PEEK cage	Spinal fusion	Meisel et al. (2008)
BMP-2	PEEK	Coated BMP-2 loaded phosphorylated gelatin on PEEK	Promote cell adhesion and proliferation, effectively promote osteogenic differentiation and improve biological activity	Wu J. et al. (2018)
BMP-2	PLA	Scaffold surface-modified with DA and BMP-2	Bone regeneration occurred in the skull defects of rats; the fibrous bone tended to connect to form continuous bone tissue	Zhang X. et al. (2019)
BMP-2	PLGA	DA and BMP-2 coatings	Significantly promoted *in vivo* bone formation in critical-sized calvarial bone defects	Ko et al. (2013)
BMP-2	PLGA	Modified the surface of the scaffold with DA	Significant promoting effect on cell adhesion and proliferation. Alkaline phosphatase activity, calcium deposition and osteogenesis are highly expressed	Zhao X. et al. (2017)
	HA			

## 4 Clinical Applications of BMPs

### 4.1 Open or Nonunion Fractures

Open or nonunion fractures are a challenging complication with often unpredictable results. They can have devastating effects on patients, often require multiple surgeries and long-term recovery, and can lead to severe psychological and functional disabilities. Treatment of open or nonunion fractures is difficult. The two main principles of treatment are internal fixation to stabilize the structure and improvement of bone biology (Singh et al., 2016; Hissnauer et al., 2017).

AGB currently exhibits the best cure rate and is the safest method for treating bone defects. At present, most clinical studies have shown that BMP can achieve a faster healing time in treatment of bone nonunion, and the combination of BMP and ABG has a more significant therapeutic effect (Flierl et al., 2013; von Ruden et al., 2016). Hissnauer et al. (2017) retrospectively studied 10 children with congenital pseudarthrosis of the tibia (CPT) or tibial nonunion treated with rhBMP-2. Nine of the ten patients achieved healing after initial surgery. rhBMP-2 may provide an appropriate option for treatment of CPT or persistent tibial nonunion in children and adolescents. In a study by Singh et al. (2016), 42 patients with refractory upper limb bone nonunion who were continuously treated with rhBMP-7 were followed up. Bmp-7 was used alone in 1 case and combined with ABG in 41 cases. Forty fractures showed both clinical and radiographic healing, while the other two patients showed partial radiographic healing. Combined treatment with ABG and rhBMP-7 has achieved results in the treatment of refractory upper limb nonunion. Therefore, combined application of rhBMP and ABG may have a better effect on promoting bone nonunion healing than BMP alone.

Current studies have shown that in the treatment of bone nonunion, although rhBMP-2 and rhBMP-7 are helpful for fracture healing, the more critical factor is the method employed during the operation, which may be the most important factor affecting postoperative fracture healing (Hackl et al., 2017; Dilogo et al., 2019). One year after rhBMP application, bone nonunion did not heal compared with the nonapplication group. Because the non-BMP group also reached the healing standard, whether there is a difference in the healing time of nonunion needs more research. Much debate remains, especially with regard to the safety and efficacy of rhBMP. At present, there are few data on adding rhBMP to the treatment for long bone nonunion, and the role and indications of rhBMP in the treatment of nonunion have not been clarified. The limitations of these studies include retrospective review, a small number of patients, and a lack of randomization. Prospective randomized controlled trials are needed to investigate the long-term efficacy and safety of rhBMP in these cases. rhBMP has been successfully used for the treatment of nonunion, and it should be warned that the use of external growth factors may bring adverse complications. However, studies have found a risk of impaired wound healing and inflammation following BMP use, but this was only a temporary problem and subsided over the course of continuous treatment, with no other complications (von Ruden et al., 2016; Hissnauer et al., 2017).

**FIGURE 8:**
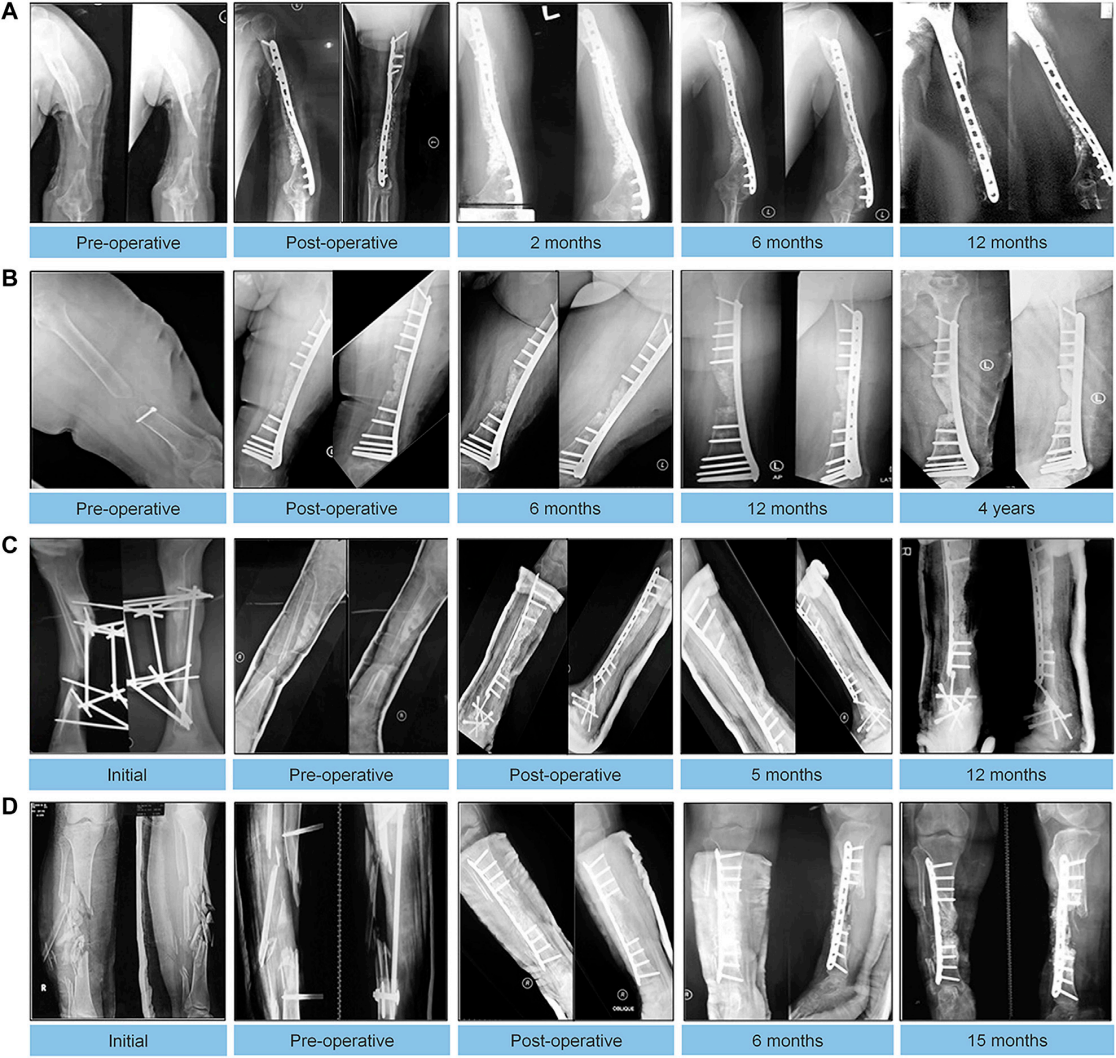
Autologous MSC implantation, hydroxyapatite, BMP-2, and internal fixation for treating critical-sized defects. Radiographic results of follow-up visits of 4 cases. **(A)**: An 18-year-old male with 5-cm bone defect of the humerus. **(B)**: An 18-year-old male with 5-cm bone defect of the humerus. **(C)**: A 28-year-old male with 7-year history of 8-cm bone defect of the right tibia. **(D)**: A 24-year-old female with 12 cm bone defect of the tibia. Reprinted with permission from (Dilogo et al., 2019).

Recent studies have shown that with the development of implant materials, a variety of new implant materials equipped with BMP can continuously stimulate local bone growth. In particular, HA has been used as a material for clinical application. BMP combined with autologous bone grafts and hydroxyapatite particles has a unique osteoinduction effect on mesenchymal cells present in autologous bone grafts and is also associated with the effect of hydroxyapatite on bone conduction at nonunion sites (Caterini et al., 2016; Dilogo et al., 2019). Caterini et al. (2016) reported 12 patients with refractory humeral nonunion who were treated with rhBMP-7, HA and ABG for nonunion. The average healing time of nonunion in all patients was 7.3 months, and function was basically restored. The patients were satisfied with the effect. This shows that the combined application of rhBMP-7 and HA is an effective method to stimulate bone healing. Dilogo et al. (2019) treated 6 patients with critical-sized defects with a combination of autologous BMSCs, HA particles, rhBMP-2 and mechanical stabilization. Follow-up for at least 12 months after surgery showed significant improvement in function in all cases (Figure 8).

rhBMP-2 and rhBMP-7 combined with other bone growth-promoting substances, such as HA and ABG, can significantly enhance the degree of fracture healing in patients. During treatment, the effects of the mutual promotion of various treatment methods should be evaluated in detail. To avoid complications, it is particularly important to find a treatment method suitable for combined application with rhBMP.

### 4.2 Vertebral Fusion

In spine surgery, nonunion is a challenging problem. Spinal instability can cause nerve damage and many sequelae, which hinder the treatment effect. Therefore, surgery is needed to achieve fusion. The traditional surgical method has a low cure rate and can cause many complications. Therefore, whether rhBMP treatment can increase the cure rate and reduce complications has an important impact on the development of spinal surgery (Meisel et al., 2008; Annis et al., 2015; Sayama et al., 2015; Stiel et al., 2018).

The gold standard for spinal fusion is a local autogenous bone graft, with autogenous iliac crest or rib bone extracted separately. However, it can lead to complications, such as pain at the site of bone removal, haematoma, infection, and increased risk of fracture. Bone nonunion remains a challenging problem. Especially in paediatric spinal surgery, too many autogenous bone grafts will bring more serious complications due to the insufficient amount of autogenous bone in children. Therefore, it is very important to find a treatment method that can replace ABG or reduce the number or amount of bone grafts. Currently, ABG combined with BMP is used to achieve spinal fusion and has received increasing attention (Singh et al., 2013; Sayama et al., 2015; Stiel et al., 2018). In one study, 13 children with spinal deformities were treated with rhBMP-2 during spinal fusion, with an average follow-up of 51 months. Analysis of clinical and radiographic results showed that 11 cases of spinal fusion were observed, with no significant increase in complications. The rate of spinal fusion in children treated with rhBMP-2 was high, and the incidence of complications was not significantly increased. Therefore, we consider recombinant human BMP-2 to be an option in paediatric spinal surgery, especially in cases of impaired bone healing due to congenital, systemic or local disease (Stiel et al., 2018). In one report, a retrospective review of 11 paediatric patients with L5-S1 neuromuscular spinal deformity treated with long segment fusion and rhBMP-2 was performed, and this treatment had the advantages of a lower complication rate, less bleeding, and shorter surgical duration compared to peripheral nerve fusion (Gressot et al., 2014). In another study, 17 patients with degenerative lumbar diseases were treated with rhBMP-2 carried in a collagen sponge. The patients were followed up for 3 and 6 months after the operation. All the patients showed signs of spinal fusion, and there were no complications, such as vertebral collapse and pain. All 17 patients reached the standard of vertebral fusion healing after 6 months (Meisel et al., 2008). In adult L5-S1 vertebral deformity surgery, BMP-2 combined with sacral internal fixation was retrospectively studied in 61 patients, and the vertebral fusion rate was 97%. A satisfactory fusion rate was obtained by combining low-dose BMP-2 with internal sacral fixation (Annis et al., 2015).

While satisfactory results have been achieved with rhBMP-2, we cannot ignore the existence of postoperative complications. The Rush University Department of Orthopaedic Surgery reviewed 573 adult patients who received rhBMP-2 during vertebral surgery. Of these patients, 91.4% achieved postoperative healing standards. However, in other cases, there were complications, and symptomatic ectopic bone formation, vertebral osteolysis, and pseudojoint complications were detected after treatment with rhBMP-2 (Singh et al., 2013). In a follow-up study of 119 patients, 33 patients received autologous iliac bone grafts, and 86 patients were treated with rhBMP-2. The data suggest that the use of rhBMP-2 reduced the incidence of donor-site complications due to autologous bone grafts but also introduced treat-site complications, including postoperative radiculitis and ectopic bone formation. The most common complication in the rhBMP-2 group was postoperative radiculitis, and hydrogel sealing significantly reduced the incidence of postoperative radiculitis (Rihn et al., 2009). Therefore, the application of hydrogel sealant and rhBMP can reduce the incidence of postoperative complications.

The general conclusion is that the use of rhBMP-2 in adult vertebral surgery can achieve satisfactory results and reduce the incidence of complications. Although some studies in adults have shown that rhBMP-2 has a positive effect on vertebral fusion, the use of rhBMP-2 in children has shown a higher failure rate of vertebral fusion than in adults. Therefore, the application of rhBMP-2 in spinal surgery in children and in spinal fusion in adults should be studied separately. BMP affects the development of the vertebral body in children, and there should be more evaluation and research.

### 4.3 Maxillofacial Bone Enhancement

Maxillofacial bone enhancement is also common in head and neck surgery. As a result of congenital dysplasia or complete or partial removal of the mandible and other facial bones after tumour surgery or trauma, extensive bone defects usually appear in the oral maxillofacial region. ABG has been used to fix these defects. In addition, bone graft materials and autologous bone grafts have been used to treat other defects, such as congenital cleft palate, facial fissures, and facial asymmetry. rhBMP has also been widely used in maxillofacial bone repair. For example, a custom-made carrier based on a patient’s unique CT scan can be designed to perfectly fill bone defects, resulting in accurate and low-dose BMP loading with minimal impact on surrounding tissue and reduced side effects (Herford and Boyne, 2008; Jensen et al., 2012; Nam et al., 2017; Park et al., 2017; Ayoub and Gillgrass, 2019).

The application of rhBMP without bone grafts in the treatment of mandibular defects has achieved a good therapeutic effect. In the treatment of 14 cases of bone defects caused by maxillofacial tumours or osteomyelitis, rhBMP-2 was applied to collagen, and no bone graft material was used. In all cases, the bones were successfully repaired in the toothless area, supporting the application of rhBMP-2 in facial bone regeneration or repair (Herford and Boyne, 2008). In another study to treat severe maxillary sinus atrophy, BMP-2 was added to the implant in 10 patients and successfully bound to bone a year later to form a stabilized prosthesis. In the case of severe maxillary atrophy, zygomatic implants with rhBMP-2 added are a viable option (Jensen et al., 2012). In a study treating medication-related osteonecrosis of the jaw (MRONJ), the efficacy of rhBMP-2 combined with leukocyte-rich and platelet-rich fibrin (L-PRF) was evaluated. The lesions completely resolved with the combination of L-PRF and rhBMP-2, which was significantly different from the results of treatment with L-PRF alone. Therefore, the additional use of rhBMP-2 significantly improved the healing of medication-related osteonecrosis of the jaw (Park et al., 2017). BMP-2 in combination with both hydroxyapatite and bovine-derived xenografts can effectively enhance the alveolar ridge in treatment of augmentation of the alveolar ridge, and BMP-2 in combination with hydroxyapatite is especially effective in repairing complex bone defects (Nam et al., 2017). The use of a collagen carrier enables rhBMP-7 to be used more effectively in surgery. Collagen can be injected directly into the cleft area of the alveolar crest, which facilitates surgery because it requires a smaller incision, thus reducing complications. This provides a guarantee of the safety of BMP-7 applied to the immature area of bone. During a follow-up period of 10 years, maxillary bone growth was similar to that of autologous bone grafts in the area where rhBMP was applied, without excessive bone fusion or excessive bone growth, and rhBMP-7 has been found to be safe in the treatment of alveolar bone defect repair in children (Ayoub and Gillgrass, 2019). Table 5 lists clinical applications of BMPs.

**TABLE 5:** Clinical applications of BMPs.

Disease	Therapies	Total number of patients	Number of effective patients	Effective rate (%)	References
CPT or persistent tibial nonunion in children and adolescents	rhBMP-2	10	9	90.0	Hissnauer et al. (2017)
Refractory upper limb bone nonunion	rhBMP-7 and ABG	41	39	95.1	Singh et al. (2016)
Refractory humeral nonunion	rhBMP-7, HA, and ABG	12	12	100	Caterini et al. (2016)
Critical-sized defect	rhBMP-2, HA, and autologous BMSCs	6	6	100	Dilogo et al. (2019)
Pediatric spinal deformity	rhBMP-2	13	11	84.6	Stiel et al. (2018)
Neuromuscular spinal deformity	rh-BMP-2, segmental spinal instrumentation system	11	10	90.9	Gressot et al. (2014)
Degenerative lumbar disease	rhBMP-2 carried by collagen sponge	17	17	100	Meisel et al. (2008)
Adult L5-S1 vertebral deformity	rhBMP-2, multi-level spinal and fusion pelvic fixation	61	59	97	Annis et al. (2015)
Vertebral disease	rhBMP-2, laminectomy with bilateral facetectomy, single TLIF cage, unilateral pedicle screw fixation	573	524	91.4	Singh et al. (2013)
Bone defects caused by maxillofacial tumors or osteomyelitis	rhBMP-2 carried by collagen sponge	14	14	100	Herford and Boyne, (2008)
Severe maxillary sinus atrophy	rhBMP-2 was added to the implant	10	10	100	Jensen et al. (2012)
Medication-related osteonecrosis of the jaws	rhBMP-2 combined with L-PRF	30	30	100	Park et al. (2017)
Unilateral cleft lip and palate	rhBMP-7	9	9	100	Ayoub and Gillgrass, (2019)

In clinical application of rhBMP, most previous retrospective studies have confirmed that rhBMP has the effect of repairing bone defects and can reduce the occurrence of complications. The use of rhBMP has led to preliminary achievements in the treatment of clinical orthopaedic diseases and has good efficacy in promoting bone healing and reducing complications. However, it should not be neglected that the efficacy of BMP alone is not significant, and there are other influencing factors in the process of bone healing. Combining other factors that can promote bone healing, such as ABG, HA particles, hydrogels, collagen sponges, and bone substitutes, with rhBMP can lead to a better effect. Clinical therapy is rarely based on a single bioactive molecule and almost always requires combinatorial approaches, since the combined use of bioactive molecules usually achieves greater efficacy. In the prospect of using BMPs in the treatment of orthopaedic diseases, the combined application of implants and BMPs is obviously a research trend. Because implants can be loaded with BMP, a control and continuous release system can be formed to achieve continuous and effective osteogenesis stimulation at the bone defect site. Further research progress in implants can solve a series of problems in the application of BMP.

## 5 Current Limitations and Future Perspectives

In orthopaedics, delayed healing or nonunion of bone caused by a large bone defect area has always been a difficult treatment problem. How to promote bone regeneration and bone healing has become an important field of research. Currently, prosthesis implantation, autogenous bone transplantation, local loading of drugs or growth factors and other methods can promote bone regeneration and bone healing. Among the growth factors that have been the focus of research in recent years, BMPs have the strongest osteogenic activity. Although there is a superficial understanding of BMPs, for example, BMPs stimulate osteoblast differentiation through the Smad pathway, the signalling pathway of BMPs is still under study. What can be determined now is that BMPs do not rely on a single signalling pathway to function; many signalling pathways work together, and various factors affect each other, thereby stimulating osteoblasts in the body to repair bone defects. Interestingly, the individual skeleton is a composite structure, and its specific growth pattern is constructed by numerous gene lineages and domains that regulate gene expression. The individual enhancer in BMP genes provides a genome to precisely control the growth of cartilage and bone so that bone growth can be individually regulated in specific parts of the body. In addition, BMP gene expression can be found not only in the skeletal system but also in other organs and tissues. This shows that the human body is a complex overall structure that is jointly regulated by the genome to maintain homeostasis and repair damage. At present, *in vitro* studies and animal studies have shown that BMPs have an important role in cartilage differentiation, bone differentiation and tendon and ligament differentiation. BMP-2, BMP-4, BMP-5, BMP-6, BMP-7 and BMP-9 have obvious stimulating effects on cartilage formation and bone healing, while BMP-12, BMP-13 and BMP-14 have significant effects on tendon and ligament repair. In addition, BMP and other cytokines also have a synergistic effect, which can enhance the biological activity of each other and achieve better osteogenesis, chondrogenesis, tendon formation and vascular formation.

In 2002, the FDA approved rhBMP-2 and rhBMP-7 for clinical treatment of issues such as vertebral fusion, open or nonunion fractures, and maxillofacial bone reinforcement. With the increase in follow-up data in recent years, BMPs have been verified to be beneficial for the treatment of diseases. However, in the process of applying BMPs, we should pay attention to preventing complications and consider individualized treatment for adults and children. The use of local high-dose BMPs may cause various complications, such as ectopic bone formation. Slow and sustained release of BMPs at a low dose will enhance bone healing and reduce the occurrence of complications. How to achieve slow and sustained release of BMPs at low doses is a difficulty that needs to be solved. At present, implantation of a scaffold with a porous structure and coating of the implant can achieve slow and continuous release of growth factors at the site of bone defects. However, implants made from a single material have various disadvantages in terms of strength, stability, durability, wear resistance, bone conductivity and biocompatibility. Implants made of composite materials can solve this problem and promote bone healing at the implantation site.

The development of bone tissue engineering provides a good solution for the application of BMP. At present, the research focus has been focused on the development of composite scaffolds, which have stronger biological functions. Importantly, with the development of various BMP delivery systems, BMP will be more reasonably loaded into the composite scaffold, so that BMP can continue to release slowly *in vivo*, and continue to stimulate osteoblast growth and induce bone formation in the process of fracture healing. Most notably, research on composite implant materials loaded with BMPs will provide better prospects for treatment of orthopaedic diseases.

## 6 Conclusion

BMPs have a powerful role in stimulating the differentiation of stem cells into bone, chondroblasts and tendons, which provides a new option for treatment of orthopaedic diseases. Application of BMP at the disease site requires a collagen sponge or other implant carrier to achieve slow and continuous release of BMP, thus achieving continuous and effective stimulation of bone healing. The selection of implant materials is particularly important for the degree of bone healing recovery. Composite materials can combine the advantages of various materials to endow implants with better biocompatibility, shape plasticity, antimicrobial properties and osteogenesis capacity. This review mainly introduces the biological function of BMP in the field of orthopaedics and presents the latest progress in implant materials equipped with BMP. Further development and modification of implants would be helpful in achieving better clinical application of BMP.
